# *Cutibacterium acnes*–Derived Extracellular Vesicles Promote Epithelial Ovarian Cancer Progression by Activating the KEAP1–NRF2 Antioxidant Pathway to Suppress Ferroptosis

**DOI:** 10.1111/1751-7915.70373

**Published:** 2026-05-11

**Authors:** Qifa Huang, Qi Chen, Wenjie Xiong, Yuexi Sun, Yuxiong Huang, Ang Dai, Jianying Chen, Xue Wu, Ying Jiang, Fen Wei, Qi Chen, Tingtao Chen

**Affiliations:** ^1^ Department of Obstetrics and Gynecology The Second Affiliated Hospital, Jiangxi Medical College, Nanchang University Nanchang Jiangxi China; ^2^ Jiangxi Key Laboratory of Molecular Medicine The Second Affiliated Hospital of Nanchang University, Nanchang University Nanchang Jiangxi China; ^3^ School of Clinical Medicine Jiangxi University of Chinese Medicine Nanchang Jiangxi China; ^4^ Department of Radiology, Nanchang People's Hospital Affiliated Hospital of Nanchang Medical College Nanchang Jiangxi China; ^5^ Jiangxi Province Key Laboratory of Bioengineering Drugs, School of Pharmacy Nanchang University Nanchang Jiangxi China; ^6^ National Engineering Research Center for Bioengineering Drugs and the Technologies Institute of Translational Medicine, Jiangxi Medical College, Nanchang University Nanchang Jiangxi China; ^7^ Queen Mary School, Jiangxi Medical College Nanchang University Nanchang Jiangxi China

**Keywords:** bacterial extracellular vesicles, *Cutibacterium acnes*, epithelial ovarian cancer, ferroptosis, redox metabolism

## Abstract

Bacterial extracellular vesicles are increasingly recognized as important mediators of microbe–host communication, yet their functional roles within tumour‐associated microbiota remain poorly understood. Here, we investigated whether extracellular vesicles derived from *Cutibacterium acnes* (CEVs) regulate host redox metabolism and ferroptosis in epithelial ovarian cancer (EOC). Using integrated in vitro and in vivo models, we found that CEVs significantly promoted tumour growth and induced transcriptional reprogramming toward antioxidant defence and ferroptosis resistance. Mechanistically, CEVs activated the KEAP1–NRF2 signalling axis through coordinated downregulation of ACSL4 and KEAP1, leading to enhanced glutathione biosynthesis, increased GPX4 activity, reduced lipid peroxidation and decreased intracellular reactive oxygen species levels. These metabolic alterations suppressed ferroptosis and promoted tumour cell survival. Importantly, pharmacological induction of ferroptosis using RSL3 abolished the tumour‐promoting effects of CEVs, demonstrating that ferroptosis suppression is essential for CEVs‐mediated tumour progression. Collectively, our findings identify bacterial extracellular vesicles as functional modulators of host redox metabolism and ferroptosis, revealing a previously unrecognized mechanism by which tumour‐associated microbiota influence cancer progression.

## Graphical abstract

*C. acnes*‐derived extracellular vesicles promote epithelial ovarian cancer progression. CEVs reprogram redox homeostasis and suppress ferroptosis by modulating key regulators.

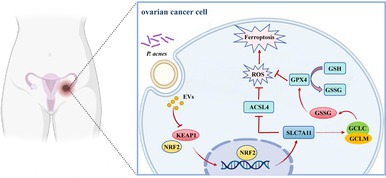

## 1 Introduction

Tumours are defined as excessive pathological tissue growths, characterized by dysregulation of the dynamic balance between cell proliferation and apoptosis (Ma et al. 2025), which leads to locally uncontrolled growth and the manifestation of malignant phenotypes (Swamydas et al. 2022). Currently, tumourigenesis and progression are recognized as complex processes driven by the synergistic effects of multiple factors, including genetic susceptibility (Kuzbari et al. 2023), epigenetic modifications (Davalos and Esteller 2023), immune evasion (Li et al. 2024), metabolic reprogramming (Liu, Zhao, et al. 2024) and environmental exposures (Cheng and Zhang 2025). Among these, the tumour microenvironment (TME) serves as a critical regulatory hub in tumour biology, playing a decisive role in malignant progression and therapeutic response (Ma et al. 2021).

The tumour microenvironment, a dynamic system comprising tumour cells, immune cells, stromal cells, extracellular matrix, signalling molecules and resident microorganisms, exerts profound influences on tumour progression and treatment response through complex interactions (Xiao and Yu 2021; Michán‐Doña et al. 2024). Traditionally, tumour tissues were regarded as sterile. However, advances in systems biology and multi‐omics approaches have revealed intratumoral microbiota as novel regulators of the TME (Rajbhandary et al. 2023). Intratumoral microbiota have been detected in various solid tumours, including pancreatic, breast and lung cancers and have been shown to participate in tumour biology through multiple mechanisms (Abbas and Tangney 2025). For instance, Geller et al. (2017) demonstrated that intratumoral Gammaproteobacteria in pancreatic cancer metabolize gemcitabine into inactive forms, thereby inducing chemoresistance and promoting tumorigenesis. Furthermore, Kong et al. (2021) reported that *Fusobacterium nucleatum* drives epithelial‐mesenchymal transition via the TLR4/CYP2J2/12,13‐EpOME signalling pathway, thereby facilitating colorectal cancer metastasis. Similarly, Jin et al. (2019) found that commensal bacteria accelerate lung cancer progression by amplifying inflammatory responses through activation of the myeloid cell‐IL‐23/IL‐1β‐γδT cell axis. Although tumour‐promoting effects of intratumoral microbiota have been validated in multiple cancer types (Galeano Niño et al. 2022), the composition and biological functions of intratumoral microorganisms in epithelial ovarian cancer (EOC) remain poorly understood. Therefore, elucidating the microbial composition of intratumoral communities in EOC and their interaction mechanisms with host cells is critical for unravelling EOC pathogenesis and developing innovative therapeutic strategies (Wu et al. 2023).

*Cutibacterium acnes* (formerly *Propionibacterium acnes* ), a Gram‐positive anaerobic opportunistic pathogen, has been detected in EOC tissues, providing clinical evidence for its potential involvement in tumour development. Our previous study further demonstrated that increased intratumoral colonization of *C. acnes* promotes tumour progression in EOC mouse models (Huang et al. 2022), underscoring its biological significance in EOC. In addition to *C. acnes*, other Gram‐positive anaerobic bacteria, such as *Clostridium* and *Peptostreptococcus* species, have also been reported to contribute to the formation of tumour‐promoting microenvironments in various cancers by modulating inflammation, immune responses and microenvironmental remodelling (Yu et al. 2017; Long et al. 2019). Interestingly, early studies showed that intratumoral injection of skin‐derived live *C. acnes* at high doses can suppress melanoma growth by enhancing Th1 immune responses (Tsuda et al. 2011). In contrast, tumour‐resident *C. acnes* in EOC naturally exists at low abundance, primarily inducing chronic inflammation and activating pro‐tumour signalling pathways (Huang et al. 2022). These observations indicate that *C. acnes* can exhibit distinct biological effects depending on its ecological context and abundance, highlighting the complexity of its functional roles. Recent studies further emphasize that bacterial extracellular vesicles (BEVs) serve as critical mediators of host–microbe interactions (Díaz‐Garrido et al. 2021; Liu, Akbar, et al. 2024). *C. acnes*–derived extracellular vesicles (CEVs) can be internalized by host cells to regulate intracellular signalling and remodel the tumour microenvironment (Choi et al. 2018; Witwer et al. 2021; Cheung et al. 2024). Unlike live bacteria, which primarily act through immune mechanisms, CEVs represent a unique pathway through which tumour‐resident *C. acnes* modulates EOC progression (Yang et al. 2021; Jingushi et al. 2024). Therefore, investigating the functions of CEVs is essential for understanding the roles of intratumoral microbes in ovarian cancer.

In this study, we isolated and characterized CEVs. in vitro, we systematically assessed their effects on ID8 cell proliferation, invasion, migration and apoptosis. To elucidate the molecular mechanisms underlying CEVs‐mediated tumour promotion, we performed RNA sequencing followed by validation using immunofluorescence, Western blotting and RT‐qPCR analyses. Finally, we confirmed the pro‐tumour effects and key signalling pathways of CEVs in vivo by intratumoral injection in an ID8‐induced EOC mouse model. The aim of this study was to elucidate the mechanism by which CEVs promote EOC progression and to provide a basis for the development of potential therapeutic strategies.

## 2 Materials and Methods

### 2.1 Isolation and Characterization of Extracellular Vesicles Derived From *Cutibacterium acnes*

*Cutibacterium acnes* was cultured anaerobically in 50 mL of brain heart infusion (BHI) medium for 48 h. After incubation, bacterial cells were harvested by centrifugation at 3000 × g for 10 min at 4°C. The resulting pellet was washed twice with sterile anaerobic phosphate‐buffered saline (PBS) and resuspended to a final concentration of 4 × 10^5^ CFU/100 μL under anaerobic conditions. To prepare for downstream applications, the suspension was heat‐inactivated in a 95°C water bath for 15 min. Subsequently, the bacterial suspension was centrifuged to collect the culture supernatant, from which CEVs were isolated following established protocols for gram‐positive bacterial EV purification (Wang et al. 2023). For functional assays, CEVs were isolated from a defined volume of bacterial culture supernatant and resuspended proportionally in PBS, such that the final CEVs preparation corresponded to the vesicular fraction derived from the same initial volume of culture supernatant used in the supernatant‐treated group. This approach was adopted to ensure comparability between treatment conditions. The protein concentration of purified CEVs was measured using the Bradford assay, and aliquots were stored at −80°C for future use.

For the characterization of CEVs, Nanoparticle Tracking Analysis (NTA) was used to assess their concentration and size distribution. CEVs samples were diluted 1:1000 in PBS to obtain an optimal particle count of 60–100 per frame, and measurements were processed using ZetaView software (v8.05.14 SP7). To examine vesicle morphology, transmission electron microscopy (TEM; Hitachi HT7700) was performed. In brief, CEVs suspensions were placed onto 200‐mesh copper grids and allowed to adsorb for 10 min. The grids were then stained with 2% phosphotungstic acid for 3 min, followed by blotting of excess stain and air‐drying. Imaging was carried out using standard TEM protocols.

### 2.2 Cell Culture and Functional Assays

To assess the effects of *Cutibacterium acnes*–derived products on ovarian cancer cell behaviour, functional assays including cell proliferation, colony formation, migration, invasion and apoptosis were performed. The ID8 ovarian cancer cell line (official name: ID8; RRID: CVCL_IU14; species: *Mus musculus* ; sex: female; tissue of origin: ovarian surface epithelium) was obtained from Shanghai Fuxiang Biotechnology (Cat# XF1030, Shanghai, China) in May 2019. Cells were cultured in Dulbecco's Modified Eagle Medium (DMEM) supplemented with 10% foetal bovine serum (FBS) and 100 U/mL penicillin–streptomycin (Solarbio, China) and maintained in a humidified incubator at 37°C with 5% CO_2_. The identity of the cell line was authenticated by the supplier using short tandem repeat (STR) profiling across 20 loci, showing a 100% match with the reference genotype. According to the International Cell Line Authentication Committee (ICLAC) database, this cell line has not been reported as misidentified or contaminated. In addition, the cells were confirmed to be free of mycoplasma contamination prior to use in the described experiments. Detailed metadata and authentication parameters for these cell lines are summarized in Table S1.

ID8 cells were subjected to the treatments specified in the experimental design. For cell viability and proliferation assays, 1 × 10^4^ cells per well were seeded in 96‐well plates and assessed at 0, 6, 24 and 48 h using the Cell Counting Kit‐8 (CCK‐8, MedChemExpress, China) and haemocytometer‐based cell counting. For colony formation assays, 1000 cells per well were plated in 6‐well plates and cultured for 7 days, followed by fixation with methanol, staining with 0.1% crystal violet and counting of visible colonies. Cell invasion was evaluated using 24‐well Transwell chambers, with cells suspended in serum‐free medium in the upper chamber and 600 μL of medium containing 10% FBS in the lower chamber as a chemoattractant; after 24 h, cells that had migrated to the lower membrane surface were fixed, stained with 0.1% crystal violet and quantified under a microscope. For migration (wound healing) assays, 6 × 10^5^ cells were seeded in 6‐well plates and grown to confluence, after which a uniform scratch was created with a sterile pipette tip; images of the wound area were captured at 0 and 24 h, and migration was quantified by measuring the distance between wound edges. Apoptosis was assessed by collecting cells, performing Annexin V/PI staining and analysing the proportion of apoptotic cells using flow cytometry.

### 2.3 RNA Sequencing and Transcriptomic Analysis

Genome‐wide gene expression analysis was performed on ID8 cells treated with PBS or CEVs (*n* = 3). RNA extraction, quality assessment, library preparation and sequencing were conducted by Shanghai PaiSeno Biotechnology Co. Ltd. Differentially expressed genes (DEGs) were identified using the criteria of *p <* 0.05 and fold change ≥ 2. Samples with biological replicates were analysed using DESeq2 and results were visualized with heatmaps. Gene Ontology (GO) and Kyoto Encyclopedia of Genes and Genomes (KEGG) enrichment analyses were performed using a hypergeometric distribution algorithm to identify significantly enriched functional categories. In addition, Gene Set Enrichment Analysis (GSEA) was conducted using the GSEA software to compare gene expression profiles between the PBS and CEVs treatment groups and to reveal potential pathway alterations.

The RNA‐seq data generated in this study have been deposited in the Sequence Read Archive (SRA) of the National Center for Biotechnology Information (NCBI) under BioProject accession number PRJNA1338457 and are publicly available.

### 2.4 Reactive Oxygen Species (ROS) Detection

Intracellular reactive oxygen species levels were evaluated using both 2′,7′‐dichlorodihydrofluorescein diacetate (DCFH‐DA) and Dihydroethidium (DHE) fluorescent probes. After the indicated treatments, cells were washed once with PBS and incubated with either 10 μM DCFH‐DA (Beyotime, China) or 5 μM dihydroethidium (DHE; Beyotime, China) at 37°C for 20 min in the dark. Following incubation, cells were washed three times with ice‐cold PBS to remove excess probe. For DCFH‐DA staining, the fluorescence intensity of dichlorofluorescein (DCF) was measured by flow cytometry using excitation/emission wavelengths of 488/525 nm. For DHE staining, oxidized ethidium fluorescence was detected using excitation/emission wavelengths of 535/610 nm. The mean fluorescence intensity was quantified to assess intracellular ROS levels.

### 2.5 Immunofluorescence

Immunofluorescence staining was conducted using Labtek 4‐chamber slides. Cells were initially fixed with 4% paraformaldehyde, then permeabilized using 0.5% Triton X‐100. To reduce background staining, nonspecific binding was blocked by incubating samples in 2% BSA at room temperature for 1 h. Primary antibodies were applied and incubated either for 1 h at room temperature or overnight at 4°C. Following washes, secondary antibodies conjugated with Alexa Fluor dyes were added at a 1:500 dilution. Nuclei were stained with DAPI (1 μg/mL). Prior to incubation, the GPX4 antibody was pre‐evaluated with a 30‐min test on fixed cells to ensure optimal reactivity.

### 2.6 Establishment of EOC Model and Assessment of Pro‐Tumour Efficacy of CEVs

Six‐week‐old female C57BL/6 mice, obtained from Beijing Crystal BioTech Co. (Beijing, China), were housed under standardized conditions and acclimated for 1 week prior to tumour induction. To establish a subcutaneous tumour model, each mouse received a right flank injection of 5 × 10^6^ ID8 cells. Animals were housed in a controlled environment with a 12‐h light/dark cycle, free access to food and water, relative humidity of 50% ± 15% and a stable temperature of 22°C ± 2°C. All experimental procedures involving animals were reviewed and approved by the Institutional Animal Care and Use Committee of Nanchang University (Approval No. NCULAE‐20221031068) and conducted in accordance with national and institutional ethical standards.

To investigate the role of CEVs in a murine EOC model, female C57BL/6 mice were randomly divided into four groups (*n* = 6 per group) and subjected to the following regimens: (1) M group: EOC model without additional treatment; (2) MA group: mice were pretreated with a broad‐spectrum antibiotic cocktail—including ampicillin (1 g/L), vancomycin (0.5 g/L), neomycin (1 g/L) and metronidazole (1 g/L)—administered via drinking water for 2 weeks prior to tumour cell inoculation. (3) MAP group: the same antibiotic regimen followed by intratumoral injection of *C. acnes* (4 × 10^5^ CFU); and (4) MAE group: the same antibiotic pretreatment followed by intratumoral injection of CEVs (50 μL of 10 μg/mL in PBS). Both *C. acnes* and CEVs were administered directly into tumours starting at Week 4 post‐inoculation, twice weekly for a total of 4 weeks. Specifically, the concentration of *C. acnes* used in the EOC mouse model was referenced from the dosage applied in previous EOC mouse studies, whereas the CEVs concentration was determined based on the data reported in a previous study of oral cancer mouse models (Chen, Gao, et al. 2024). During the course of the study, body weight and tumour dimensions were recorded every other day using a digital balance and callipers. Tumour volume was estimated using the formula: 0.5 × length × width^2^. On Day 65, mice were humanely sacrificed, and tumours were harvested, weighed and processed. Resected tumour tissues were either immersed in 4% paraformaldehyde for subsequent histological and immunohistochemical analyses or promptly snap‐frozen in liquid nitrogen and preserved at −80°C for later molecular investigations.

To elucidate the molecular mechanisms by which CEVs influence EOC progression in vivo, C57BL/6 mice were randomly assigned to six experimental groups (*n* = 6 per group) with the following treatments: (1) M group: EOC model; (2) MA group: administration of a mixed antibiotic cocktail for 2 weeks prior to tumour implantation; (3) MAP group: same antibiotic pretreatment followed by intratumoral injection of *C. acnes* (4 × 10^5^ CFU); (4) MAE group: same antibiotic pretreatment followed by intratumoral injection of CEVs (50 μL of 10 μg/mL in PBS); (5) MAPR group: antibiotic pretreatment plus *C. acnes* injection and intratumoral administration of RSL3 (50 mg/kg); (6) MAER group: antibiotic pretreatment plus CEVs injection and intratumoral RSL3 treatment. Both *C. acnes* and CEVs were administered intratumorally twice weekly beginning at Week 4 post‐tumour implantation and continued for 4 weeks. RSL3 was delivered intratumorally three times per week throughout the same period. On Day 65, the mice were euthanized, and tumour tissues were collected for analysis following the previously established protocols. In addition, tumour growth, body weight and survival time were monitored throughout the experiment. Specific chemical information, along with the classification, characteristics and treatment regimens of the different mouse groups, are provided in Tables S2 and S3.

### 2.7 RT‐qPCR

Total RNA was extracted from cultured cells or tumour tissues using TRIzol reagent, and RNA purity and concentration were determined using a NanoDrop 2000 spectrophotometer (Thermo Fisher Scientific, USA). To eliminate genomic DNA contamination, cDNA synthesis was carried out using the PrimeScript RT Reagent Kit with gDNA Eraser (Takara). RT‐qPCR was conducted on a ViiA 7 Real‐Time PCR System (Applied Biosystems, USA) using Hieff qPCR SYBR Green Master Mix (11202ES08, Yeasen, China). The thermal cycling conditions included an initial denaturation at 95°C for 5 min, followed by 40 cycles of 95°C for 10 s, 60°C for 20 s, and 72°C for 20 s. Relative gene expression was quantified using the 2^−ΔΔCt^ method. Primer sequences are provided in Table S4.

### 2.8 Western Blot

Cells and tumour tissues were lysed using RIPA buffer containing protease and phosphatase inhibitors. After lysis, samples were centrifuged at 12,000 × g for 15 min to remove debris, and the supernatants were collected for protein quantification via a BCA assay kit. Equal amounts of total protein were separated on 10% SDS‐PAGE gels and transferred onto PVDF membranes. Membranes were then blocked and incubated overnight at 4°C with primary antibodies, followed by a 1‐h incubation at room temperature with HRP‐conjugated secondary antibodies. Protein signals were detected using the Bio‐Rad ChemiDoc XRS+ system, and band intensities were quantified using ImageJ software. Detailed information regarding the antibodies used is listed in Table S5.

### 2.9 Detection of Oxidative Stress Parameters

Oxidative stress levels in ID8 cells and mouse tumour tissues were assessed using commercial assay kits according to the manufacturers' instructions. Total antioxidant capacity and GSH/GSSG levels were measured using kits from Beyotime Biotechnology (China) (total antioxidant capacity, cat# S0119; GSH/GSSG, cat# S0053). Glutathione peroxidase (GSH‐Px) and superoxide dismutase (SOD) activities were measured using kits from Solarbio (China) (GSH‐Px, cat# S0058; SOD, cat# S0109). All assays were performed strictly following the manufacturer's protocols to ensure accurate evaluation of oxidative stress and antioxidant capacity in the samples.

### 2.10 TUNEL Assay

Tissue sections were first permeabilized with proteinase K and then incubated in a labeling solution containing biotin‐labelled deoxyuridine triphosphate (dUTP) and terminal deoxynucleotidyl transferase (TdT). Following this, the sections were reacted with FITC‐conjugated streptavidin for 1 h at 37°C and counterstained with DAPI in the dark. Fluorescence microscopy was used to visualize the samples. Cells displaying green fluorescence were considered TUNEL‐positive, indicating apoptosis. The apoptosis index (%) was determined by calculating the proportion of TUNEL‐positive cells relative to the total cell count.

### 2.11 Haematoxylin–Eosin (H&E) Staining and Immunohistochemistry (IHC)

Tumour specimens were collected, fixed in 4% paraformaldehyde and subsequently processed through a dehydration series before being embedded in paraffin. Longitudinal tissue sections, each 5 μm thick, were then obtained using a Leica CM1850 microtome for further histological analysis. These sections were then stained with H&E and mounted on glass slides. Morphological evaluation was conducted under a light microscope according to standard histological procedures.

For immunohistochemistry, paraffin‐embedded tumour sections were first deparaffinized and exposed to antigen retrieval. Following this, the slides were incubated with primary antibodies targeting Ki67, GPX4 or 4‐hydroxynonenal (4‐HNE). After rinsing with PBS, HRP‐conjugated secondary antibodies were applied for 50 min. Signal visualization was performed using 3,3′‐diaminobenzidine (DAB) as the chromogen. The sections were then counterstained, dehydrated and coverslipped. Microscopic images were captured with an Olympus IX71 microscope. Staining intensity and distribution were evaluated using the German semi‐quantitative scoring system.

### 2.12 Statistical Analysis

Data processing, figure preparation and statistical analyses were performed using GraphPad Prism version 9.0 (GraphPad Software, San Diego, CA, USA). Normally distributed continuous variables were reported as mean ± standard deviation (SD) and compared using one‐way ANOVA followed by Tukey's multiple comparison test. For data not conforming to a normal distribution, results were presented as median with interquartile range (IQR), and intergroup differences were assessed using the Kruskal–Wallis test with Dunn's post hoc analysis. Normality was evaluated using both the Shapiro–Wilk and D'Agostino–Pearson omnibus tests. Kaplan–Meier survival curves were constructed to estimate overall survival, with time‐to‐event defined as the interval from tumour inoculation to death. Mice that remained alive at the end of the observation period were treated as censored. Differences between groups were analysed using the log‐rank (Mantel–Cox) test (two‐sided). Survival analyses were performed using GraphPad Prism (version 9.0, GraphPad Software, San Diego, CA, USA). A *p* value < 0.05 was considered statistically significant.

## 3 Results

### 3.1 Identification and Characterization of Pro‐Tumorigenic Components in *C. acnes*

**FIGURE 1:**
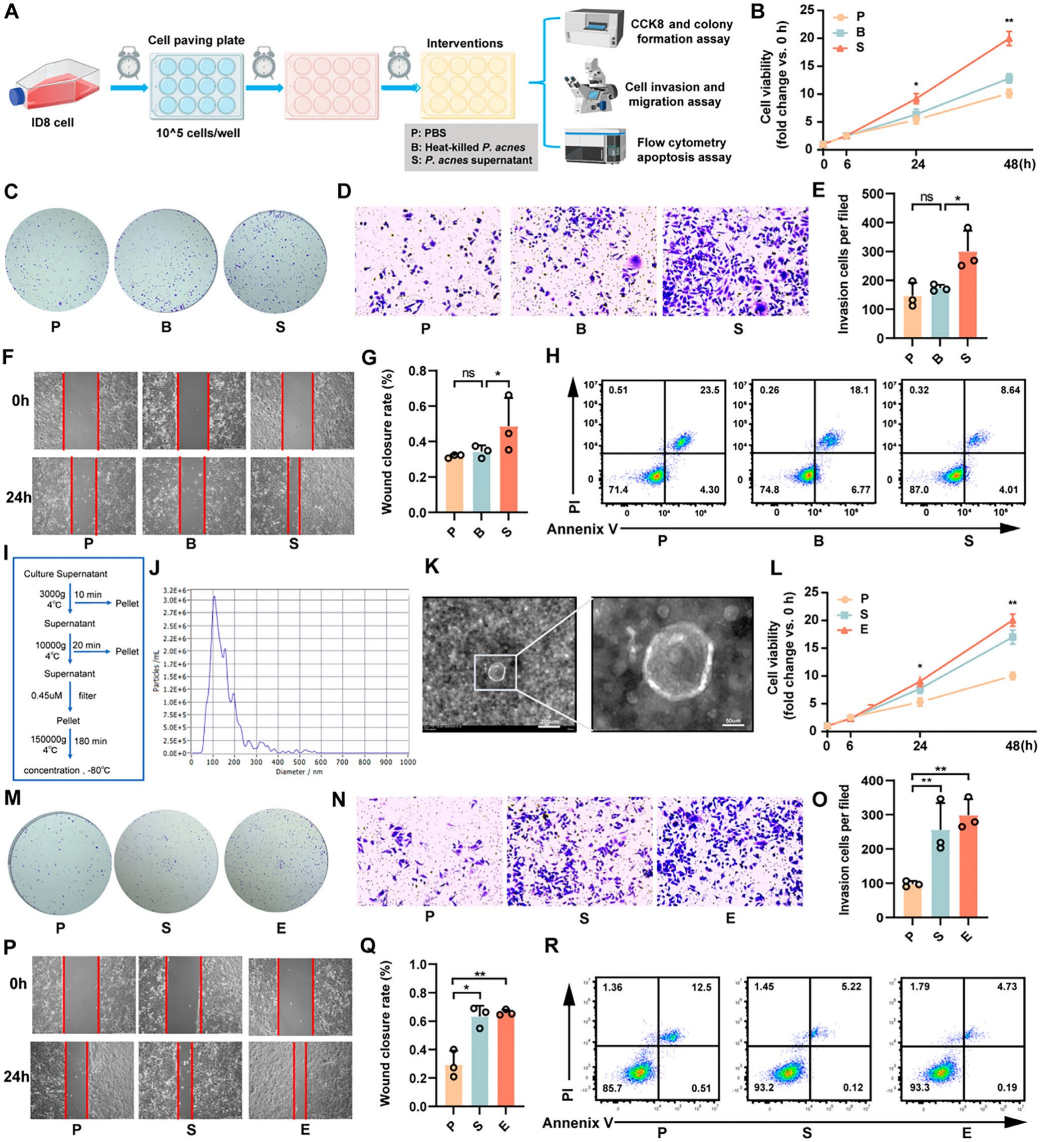
Identification and characterization of pro‐tumorigenic components in *C. acnes*. (A) Schematic representation of the overall cell experiment workflow. (B) ID8 cells were treated with PBS (P), *C. acnes* (B), or *C. acnes* culture supernatant (S), and cell viability was measured by CCK‐8 assay at 0, 6, 24 and 48 h. Data are expressed as fold change relative to the corresponding 0 h value for each group. (C) Colony formation assays were performed to evaluate the clonogenic capacity of ID8 cells. (D–G) Cellular invasion and migration were determined by Transwell and wound‐healing assays under the indicated conditions. (H) Apoptosis of ID8 cells was quantified by flow cytometry 48 h after treatment. (I) Schematic diagram depicting the ultracentrifugation‐based protocol for isolating *C. acnes*–derived extracellular vesicles (CEVs). (J) Size distribution profile of CEVs determined by nanoparticle tracking analysis. (K) Representative transmission electron microscopy image showing the typical morphology of CEVs. Enlarged views of the selected regions are presented at original magnifications ×100 and ×400, with scale bars of 200 μm and 50 μm, respectively. (L–M) CCK‐8 and colony formation assays revealed that CEVs treatment significantly enhanced ID8 cell proliferation and clonogenicity. (N–Q) Transwell and wound‐healing assays demonstrated that CEVs treatment promoted ID8 cell invasion and migration. (R) Flow cytometry analysis showed that CEVs treatment markedly reduced apoptosis in ID8 cells. Experimental groups: P (PBS), S (*C. acnes* culture supernatant) and E (CEVs). Data are presented as mean ± SD; *p <* 0.05, *p <* 0.01.

Our previous research has indicated a positive correlation between the abundance of *C. acnes* and ovarian carcinogenesis (Huang et al. 2022). To further identify the functional components of *C. acnes*, we separately prepared bacterial culture supernatant and heat‐inactivated bacterial cells, followed by co‐incubation with ID8 cells (Figure 1A). Compared with untreated controls or cells treated with heat‐inactivated bacteria, the *C. acnes* supernatant significantly promoted ID8 cell proliferation (Figures 1B and S1A,B) and enhanced colony formation capacity (Figure 1C). Further analysis using Transwell invasion assays, scratch migration assays and flow cytometric apoptosis assessment revealed that the supernatant markedly increased ID8 cell migration and invasion while inhibiting apoptosis (Figures 1D–H and S1C). These results indicate that soluble factors secreted by *C. acnes* enhance the proliferative, migratory, and invasive capacities of EOC cells, providing evidence for its tumour‐promoting role in ovarian carcinogenesis.

Given previous reports implicating EVs in the pathogenesis of multiple diseases, we hypothesized that EVs present in the *C. acnes* supernatant might be key mediators of the observed tumour‐promoting effects (D'Angelo et al. 2019). To investigate this, we isolated EVs from *C. acnes* culture supernatant using a protocol adapted from established methods for *Staphylococcus aureus* –derived EVs (Wang et al. 2023) (Figure 1I). NTA revealed that the isolated CEVs ranged in size from 30 to 600 nm, with an average diameter of 106.50 ± 0.89 nm (Figure 1J). Transmission and cryo‐electron microscopy confirmed the typical spherical morphology and lipid bilayer membrane structure of these vesicles (Figure 1K), consistent with previously reported bacterial EVs (Jain et al. 2024).

To directly assess the functional impact of CEVs on ovarian cancer cells, ID8 cells were incubated with isolated CEVs. The results showed that both the CEVs‐treated group and the culture supernatant group exhibited significantly enhanced cell proliferation and colony formation compared with the PBS control. Further analysis indicated that the CEVs‐treated group displayed slightly higher proliferation and colony‐forming capacity than the culture supernatant group, although the differences did not reach statistical significance (Figure 1L,M). In addition, the CEVs‐treated group showed a trend toward increased migration and invasion, as well as reduced apoptosis, relative to the culture supernatant group (Figures 1N–R and S2A). Collectively, these findings demonstrate that CEVs can significantly enhance the proliferative, migratory, and invasive capacities of EOC cells in vitro, suggesting a potential tumour‐promoting role in ovarian cancer progression.

### 3.2 Extracellular Vesicles Derived From *Cutibacterium acnes* Induce Transcriptional Reprogramming and Suppress Ferroptosis in ID8 Ovarian Cancer Cells

**FIGURE 2:**
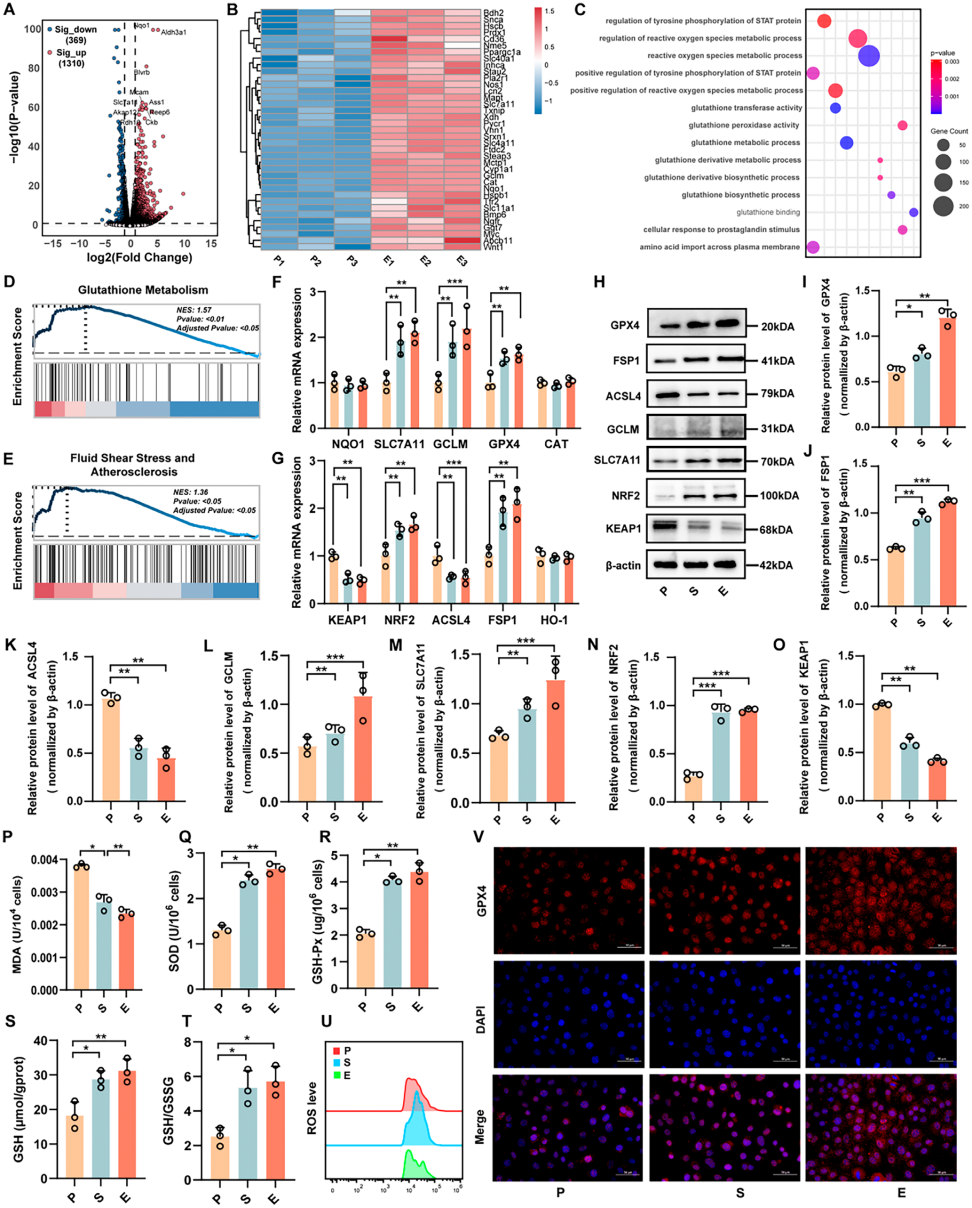
Extracellular vesicles derived from *Cutibacterium acnes* induce transcriptional reprogramming and suppress ferroptosis in ID8 ovarian cancer cells. (A) Volcano plot showing differentially expressed genes between PBS‐treated (P) and CEVs‐treated (E) ID8 cells (*p <* 0.05, fold change ≥ 2). (B) Heatmap of representative ferroptosis‐related genes significantly altered by CEVs treatment. (C) Gene Ontology (GO) enrichment analysis identifying significantly enriched biological processes, with ROS and glutathione metabolic processes being prominently represented. (D–E) Gene set enrichment analysis (GSEA) results showing significant enrichment of glutathione metabolism and fluid shear stress/atherosclerosis pathways in the CEVs‐treated group. (F–G) Quantitative RT‐PCR analysis of ferroptosis‐related genes, demonstrating upregulation of SLC7A11, GCLM, GPX4, NRF2 and FSP1 and downregulation of KEAP1 and ACSL4 following CEVs treatment (*n* = 3). (H–O) Western blot analysis confirming corresponding changes in protein expression of ferroptosis regulators and antioxidant defence components (*n* = 3). (P–U) Evaluation of oxidative stress–related indicators showing markedly reduced levels of MDA and intracellular ROS, accompanied by increased activities of SOD and GSH‐Px, elevated GSH content and a higher GSH/GSSG ratio in CEVs‐treated cells, collectively indicating an enhanced antioxidant capacity (*n* = 3). (V) Immunofluorescence staining demonstrating increased GPX4 expression in CEVs‐treated ID8 cells (scale bar = 50 μm). Data are presented as means ± SD; *p < 0.05, **p < 0.01, ***p < 0.001.

To investigate the molecular effects of CEVs on ovarian cancer cells, RNA sequencing and bioinformatics analysis were performed on CEVs‐treated ID8 cells. Principal component analysis (PCA) revealed a clear separation between the transcriptomic profiles of the PBS (P) and CEVs (E) groups (Figure S2B), indicating substantial differences in gene expression. Differential expression analysis identified 1310 upregulated and 369 downregulated genes in the E group compared with the P group (*p <* 0.05, fold change ≥ 2; Figures 2A and S2C). Notably, several ferroptosis regulators, including *Slc4a11*, *Slc7a11*, *Slc11a11*, *Gclm*, *Cat* and *Nqo1*, were significantly altered following CEVs treatment (Figures 2B and S2D,E), suggesting that CEVs may modulate ferroptosis pathways. Gene Ontology (GO) enrichment analysis identified 14 significantly enriched biological processes (Figure 2C), among which ROS metabolic process and glutathione metabolic process were prominently enriched. KEGG and gene set enrichment analysis (GSEA) further demonstrated that CEVs treatment significantly upregulated glutathione metabolism and fluid shear stress and atherosclerosis pathways (Figures 2D,E and S2F). These findings indicate that CEVs induce broad transcriptional reprogramming, particularly affecting redox homeostasis and ferroptosis.

To validate the transcriptomic findings, qRT‐PCR was performed to examine the expression of ferroptosis‐related genes. The results showed that GPX4, FSP1, GCLM, SLC7A11 and NRF2 mRNA levels were significantly increased, whereas ACSL4 and KEAP1 expression was decreased (Figure 2F,G). Western blot analysis confirmed similar trends at the protein level (Figure 2H–O). Given that CEVs treatment markedly impacted oxidative stress in ID8 cells, we further evaluated key oxidative stress markers, including MDA, SOD, GSH‐Px, reduced GSH, the GSH/GSSG ratio and intracellular ROS levels. CEVs‐treated cells exhibited significantly decreased MDA levels and intracellular ROS, accompanied by increased SOD activity, GSH‐Px activity, total GPx activity, reduced GSH levels, and an elevated GSH/GSSG ratio (Figures 2P–U and S2G), indicating enhanced cellular antioxidant capacity and reduced oxidative stress. Immunofluorescence analysis further confirmed upregulation of GPX4 expression (Figure 2V). Collectively, these results demonstrate that CEVs suppress ferroptosis by modulating ferroptosis‐related genes, enhancing antioxidant defences and inhibiting ROS accumulation, which may contribute to their tumour‐promoting effects in ID8 cells.

To further determine whether activation of the NRF2 pathway is functionally required for the CEVs‐induced anti‐ferroptotic programme, ID8 cells were treated with the NRF2 inhibitor ML385 in the presence or absence of CEVs. As shown in Figure S3A–G, CEVs treatment significantly increased the mRNA expression of GPX4, FSP1, GCLM, SLC7A11 and NRF2, while decreasing ACSL4 expression; these changes were markedly attenuated by ML385. In the EM group, the upregulation of GPX4, FSP1, GCLM, SLC7A11 and NRF2 was clearly compromised compared with the CEVs group, whereas the suppressive effect of CEVs on ACSL4 was partially reversed. Consistent trends were also observed at the protein level by western blot analysis (Figure S3H–N), further supporting that the anti‐ferroptotic gene programme induced by CEVs depends, at least in large part, on NRF2 activity.

**FIGURE 3:**
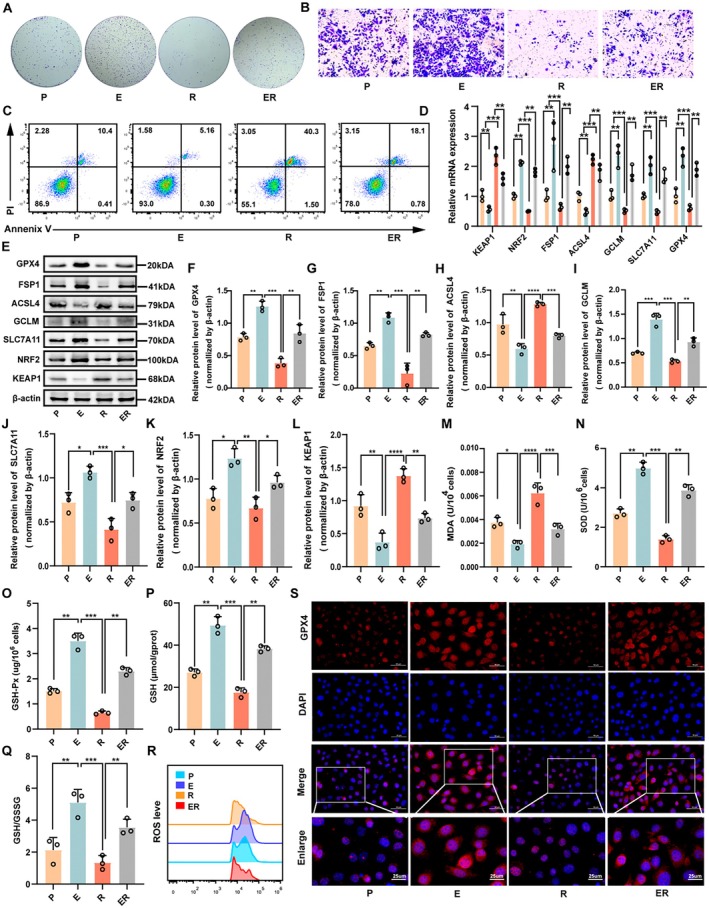
Induction of ferroptosis mitigates CEVs‐induced progression of EOC cells in vitro. (A) Colony formation assays showing that CEVs treatment markedly enhanced the clonogenic capacity of ID8 cells, whereas co‐treatment with RSL3 largely reversed this effect. (B, C) Transwell invasion assays and flow cytometric apoptosis analysis demonstrating that CEVs significantly promoted cell invasion and suppressed apoptosis, both of which were attenuated upon RSL3 treatment. (D–L) qRT‐PCR and Western blot analyses of key ferroptosis‐related genes and proteins. CEVs treatment upregulated SLC7A11, GCLM, GPX4, NRF2 and FSP1, while downregulating ACSL4 and KEAP1; these molecular changes were reversed by RSL3, indicating a shift toward a ferroptotic phenotype (*n* = 3). (M–R) Biochemical analyses demonstrating that RSL3 treatment markedly elevated MDA levels and intracellular ROS, while significantly reducing SOD activity, GSH‐Px activity, GSH content, and the GSH/GSSG ratio, collectively indicating aggravated oxidative stress compared with CEVs treatment alone (*n* = 3). (S) Immunofluorescence analysis confirming a pronounced reduction in GPX4 expression in the RSL3‐treated group (scale bar = 50 μm). Experimental groups: P (PBS), E (CEVs), R (RSL3), and ER (CEVs + RSL3). Data are presented as mean ± SD; *p < 0.05, **p < 0.01, ***p < 0.001.

We then investigated whether ferroptosis suppression functionally contributes to the tumour‐promoting effects of CEVs using RSL3, a specific GPX4 inhibitor, followed by functional assays (Figure S4A). CEVs treatment markedly promoted ID8 cell proliferation and enhanced colony‐forming capacity (Figures 3A and S4B). Transwell invasion assays, wound‐healing assays, and flow cytometric apoptosis analysis further revealed that CEVs significantly increased cell migration and invasion while suppressing apoptosis. Notably, these tumour‐promoting effects were largely reversed by RSL3 treatment (Figures 3B,C and S4C–E). To validate ferroptosis‐related molecular alterations, we examined the expression of key genes and proteins involved in the ferroptosis pathway. qRT‐PCR and Western blot analyses showed that RSL3 treatment significantly downregulated the expression of GPX4, FSP1, GCLM, SLC7A11 and NRF2, while upregulating ACSL4 and KEAP1, indicating a shift toward a ferroptotic phenotype (Figure 3D–L). We next assessed oxidative stress markers and intracellular ROS levels. RSL3 treatment significantly elevated MDA levels, reduced GSH‐Px activity, GSH and the GSH/GSSG ratio, and was accompanied by a marked increase in intracellular ROS levels (Figures 3M–R and S4F), suggesting enhanced oxidative stress. Immunofluorescence analysis further confirmed a pronounced reduction in GPX4 expression in the RSL3‐treated group (Figure 3S). Collectively, these results validate the critical role of the ferroptosis pathway in mediating the tumour‐promoting effects of CEVs and indicate that CEVs promote ID8 cell proliferation, migration and invasion by suppressing ferroptosis.

### 3.3 Extracellular Vesicles Derived From *Cutibacterium acnes* Facilitate EOC Progression in Mice by Modulating the Ferroptosis Signalling Pathway

**FIGURE 4:**
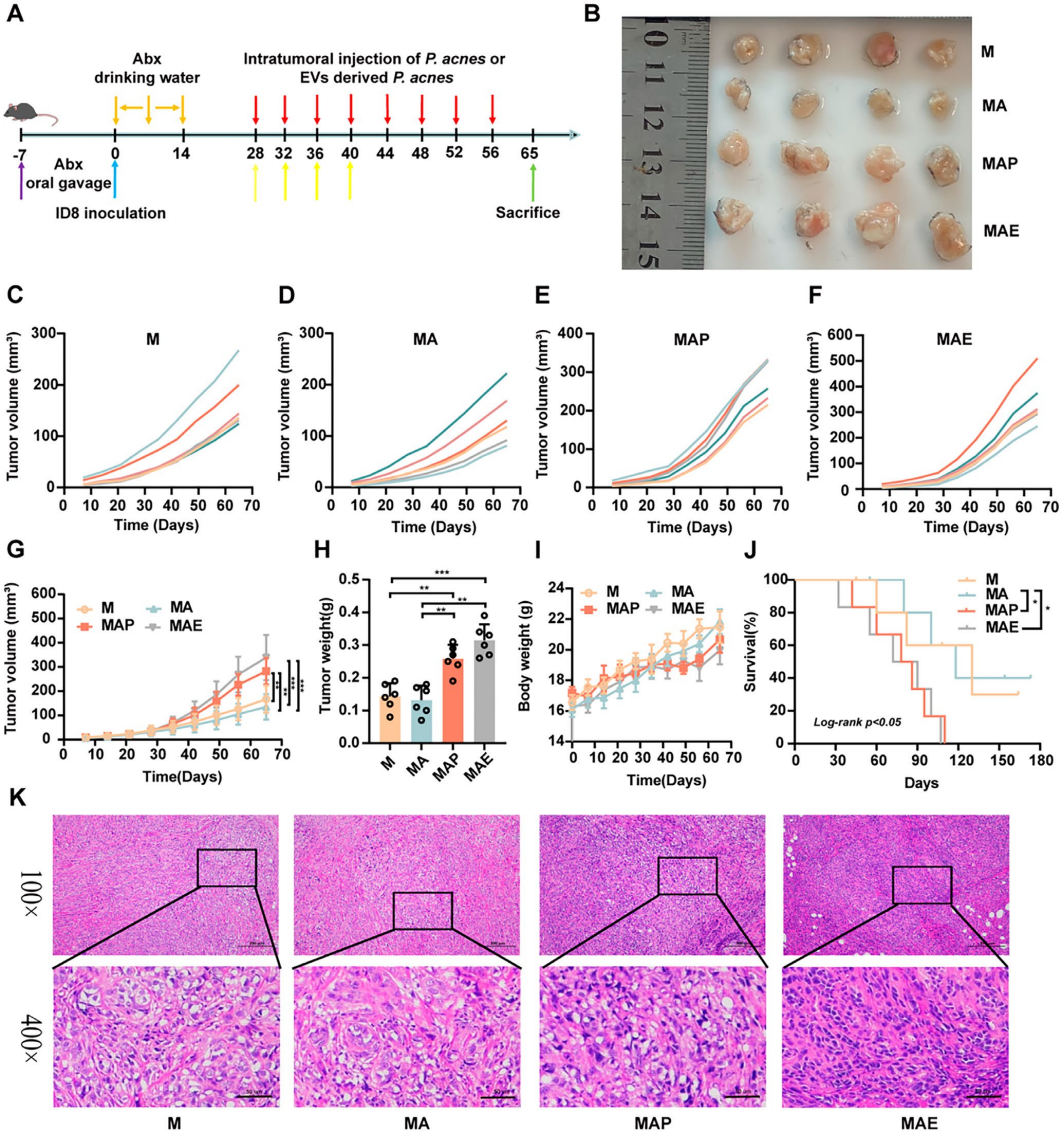
Extracellular vesicles derived from *Cutibacterium acnes* promote tumour growth and reduce survival similarly to *C. acnes* in EOC mice. (A) Schematic illustration of the in vivo experimental timeline and treatment regimen. (B) Representative tumour images collected at the study endpoint from each treatment group. (C–F) Tumour growth curves of individual mice in each experimental group (*n* = 6). (G) Average tumour growth kinetics of each experimental group over time. (H) Final tumour weights compared among groups (*n* = 6). (I) Time‐course analysis of body weight changes in mice (*n* = 6). (J) Kaplan–Meier survival curves comparing overall survival among treatment groups (*n* = 6). (K) Histological evaluation of tumour sections via H&E staining. Enlarged views of selected regions are shown in the second row (original magnification ×100 and ×400; scale bars = 200 μm and 50 μm, respectively). Experimental groups: M (EOC model), MA (antibiotic pretreatment before tumour induction), MAP (intratumoral *C. acnes* injection post‐modelling), and MAE (intratumoral CEVs injection post‐modelling). Data are presented as mean ± SD. Two‐way repeated measures ANOVA with Tukey's post hoc test for tumour volume and weight data; log‐rank test for survival data. *p < 0.05, **p < 0.01, ***p < 0.001.

To validate these in vitro results, we developed a mouse EOC model and administered a combination of antibiotics to the mice via drinking water to effectively eradicate both gut and tumour microbiota. Subsequently, *C. acnes* and CEVs were intratumorally injected into the mice, respectively (Figure 4A). To further assess whether *C. acnes* could be detected in tumour tissues following bacterial administration, we performed qPCR analysis on tumour samples. As shown in Figure S5A, the *C. acnes*‐treated group exhibited a markedly increased *C. acnes* DNA signal in tumour tissues compared with the other groups, suggesting that *C. acnes* can enter and be present within tumour tissues after administration. Consistent with this, intratumoral injection of *C. acnes* increased tumour volume compared with the MA group, although this effect was less pronounced than that induced by intratumoral injection of CEVs (Figure 4B–G). In addition, tumour weight in the EVs‐treated group was higher than that in the MA group (Figure 4H). No significant differences in body weight were observed among the groups (*p* > 0.05, Figure 4I), whereas CEVs treatment significantly reduced mouse survival compared with the MA group (*p <* 0.05, Figure 4J). Moreover, H&E staining showed that tumours from the MAE group exhibited increased cell density and a more uniform, hyperchromatic morphology relative to controls (Figure 4K).

**FIGURE 5:**
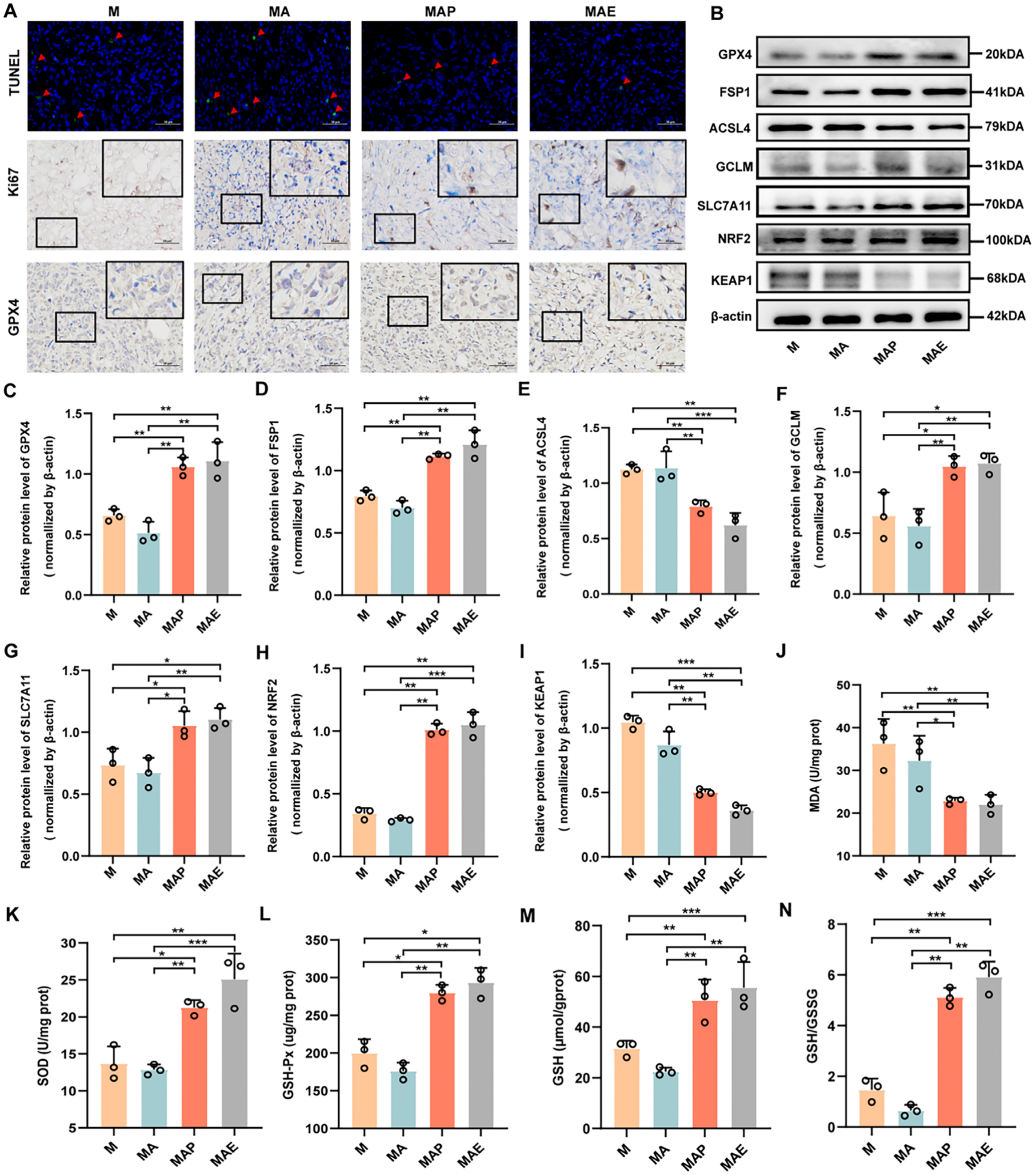
Extracellular vesicles derived from *Cutibacterium acnes* enhance EOC advancement by modulating ferroptosis. (A) Representative staining of tumour tissues: Top panel shows TUNEL staining indicating apoptotic cells; middle panel depicts Ki67 immunohistochemical (IHC) staining to assess proliferative activity; bottom panel displays GPX4 IHC staining to evaluate ferroptosis‐related protein expression. (B) Western blot analysis of ferroptosis‐associated proteins GPX4, ACSL4, FSP1, KEAP1, GCLM, SLC7A11 and NRF2 in tumour tissues; β‐actin served as a loading control (*n* = 3). (C–I) Densitometric quantification of protein expression levels using ImageJ for GPX4 (C), ACSL4 (D), FSP1 (E), KEAP1 (F), NRF2 (G), GCLM (H) and SLC7A11 (I) (*n* = 3). (J–N) Quantification of oxidative stress markers in tumour tissues, including MDA (J), SOD activity (K), GSH‐Px activity (L), reduced glutathione (GSH) levels (M) and the GSH/GSSG ratio (N). Data are presented as mean ± SD. *p < 0.05, **p < 0.01, ***p < 0.001.

To explore underlying mechanisms, TUNEL and IHC assays were performed. TUNEL staining showed markedly fewer apoptotic cells in the MAE group, suggesting resistance to apoptosis in CEVs‐treated tumours. IHC analysis further demonstrated a significant upregulation of Ki67 in the MAE group, indicating elevated proliferative activity. Additionally, CEVs exposure increased GPX4 expression, as evidenced by IHC (Figure 5A). Western blot analyses of ferroptosis‐associated proteins revealed that CEVs markedly elevated the expression of GPX4, FSP1, GCLM, SLC7A11 and NRF2, while significantly reducing ACSL4 and KEAP1 levels (*p <* 0.05; Figure 5B–I). To comprehensively evaluate the oxidative stress status, we measured multiple key indicators, including MDA, SOD, GSH‐Px, reduced GSH, the GSH/GSSG ratio and intracellular ROS levels. CEVs treatment significantly reduced MDA levels, while markedly enhancing the activities of SOD and GSH‐Px, as well as the levels of reduced GSH and the GSH/GSSG ratio (*p <* 0.01; Figure 5J–N), indicating attenuation of lipid peroxidation and enhancement of antioxidant defence capacity. Building upon these findings, immunohistochemical staining for 4‐HNE was performed to further assess lipid peroxidation at the histological level. As shown in Figure S5B,C, CEVs treatment markedly reduced 4‐HNE levels compared with the MA group. As a well‐recognized end‐product of lipid peroxidation, this reduction provides additional histological evidence that CEVs suppress lipid peroxidation and ferroptosis‐associated oxidative damage in vivo. Taken together, these findings demonstrate that CEVs suppress ferroptosis and enhance the antioxidant defence system in tumour tissues, thereby promoting cell survival and proliferation. These results suggest that the pro‐tumorigenic effects of CEVs are at least partly mediated by the modulation of oxidative stress and ferroptosis.

### 3.4 Induction of Ferroptosis Suppresses the EOC Progression Caused by CEVs In Vivo

**FIGURE 6:**
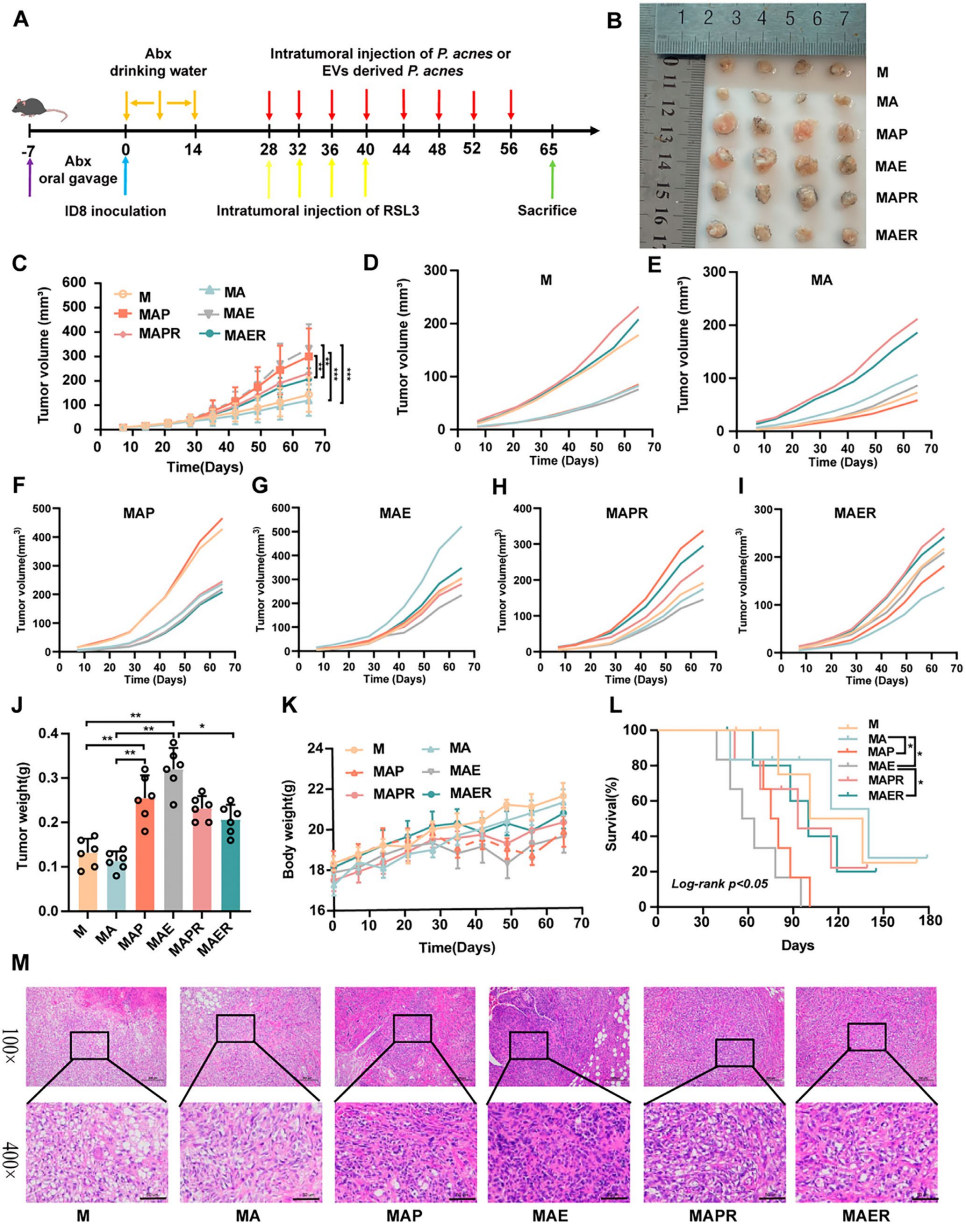
Effects of RSL3‐induced ferroptosis signalling on EOC tumour in vivo. (A) Diagram depicting the animal experimental protocol used to evaluate treatment effects. (B) Representative tumour images from each group captured 65 days after treatment initiation. (C) Tumour volume progression curves over the course of the experiment across all treatment groups. (D–I) Tumour growth curves of individual mice in each experimental group (*n* = 6). (J) Final tumour weights measured at the endpoint. (K) Body weight trends of mice recorded throughout the study period to assess systemic effects. (L) Kaplan–Meier survival curves comparing overall survival among treatment groups. (M) H&E‐stained sections of tumour tissues for histological evaluation. Enlarged images in the second row correspond to boxed areas in the first row (magnifications: ×100 and ×400; scale bars = 200 μm and 50 μm, respectively). Treatment groups: M (EOC model), MA (pretreatment with mixed antibiotics), MAP (intratumoral injection *C. acnes* after modelling), MAE (intratumoral injection CEVs after modelling), MAPR (intratumoral injection *C. acnes* meanwhile RSL3 after modelling), MAER (intratumoral injection CEVs meanwhile RSL3 after modelling). Data are expressed as mean ± SD. Tumour volume and weight data were analysed using two‐way repeated measures analysis of variance, followed by Tukey's multiple comparison test, while survival data were assessed using the log‐rank test; *p < 0.05, **p < 0.01.

To further substantiate the tumour‐promoting role of the ferroptotic pathway in EOC, we employed RSL3, a ferroptosis inducer, in combination with intratumoral injections of *C. acnes* and CEVs (Figure 6A). In line with previous findings, both *C. acnes* and CEVs significantly increased tumour volume and weight in EOC‐bearing mice. Notably, co‐treatment with RSL3 partially attenuated these tumour‐promoting effects, although tumour size and weight remained elevated compared to the antibiotic‐only (MA) group (*p <* 0.05; Figure 6B–J). Moreover, mice receiving RSL3 exhibited improved body weight maintenance and extended survival compared to those treated with *C. acnes* or CEVs alone (Figure 6K,L).

**FIGURE 7:**
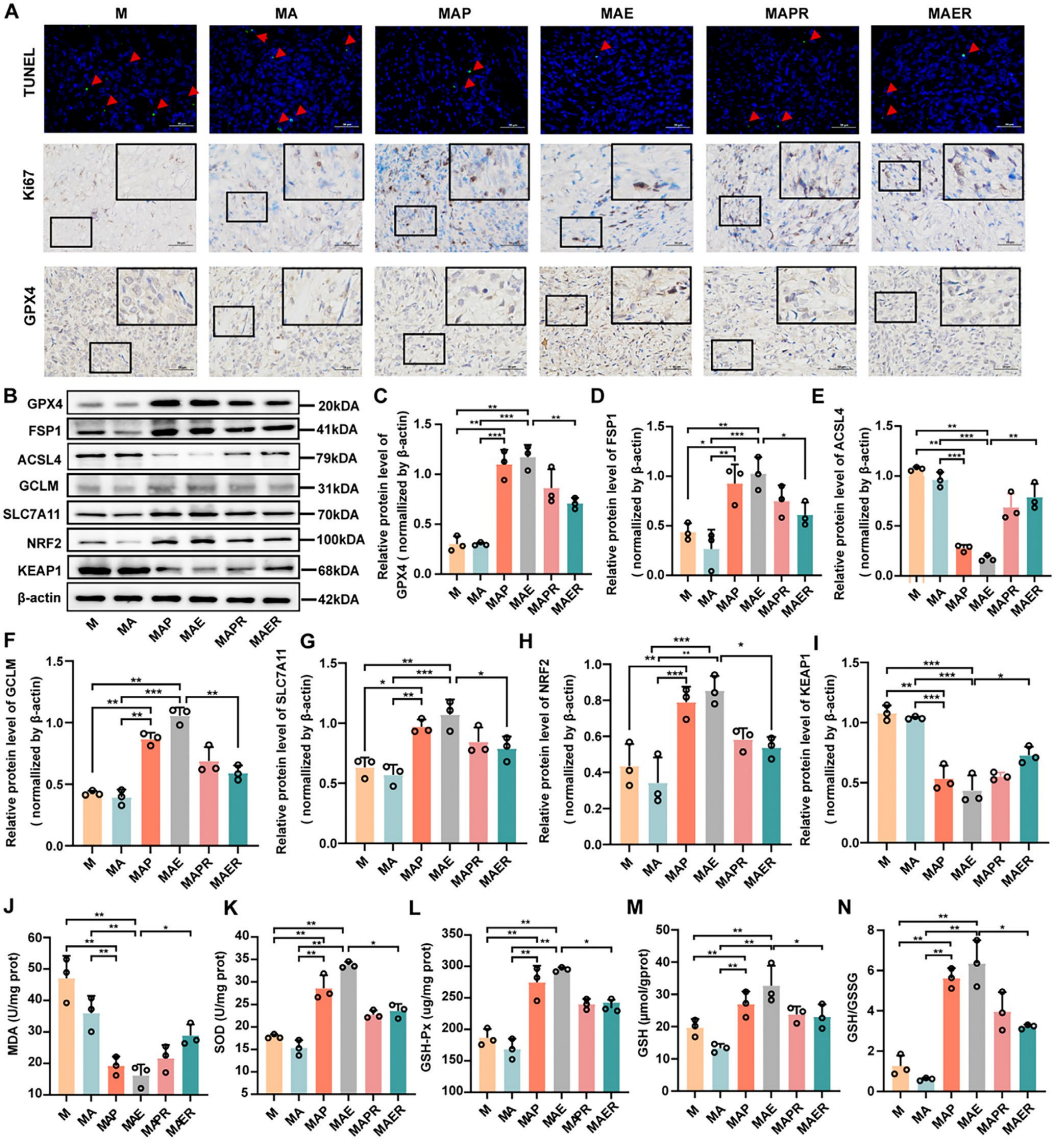
RAS‐selective lethal 3 reactivates ferroptosis and enhances oxidative stress to counteract CEVs‐induced tumour progression in vivo. (A) Representative staining of tumour sections showing apoptotic and proliferative activity. Top panel: TUNEL assay indicating apoptotic (TUNEL‐positive) cells; middle panel: Ki67 IHC staining reflecting cell proliferation; bottom panel: GPX4 IHC staining, a ferroptosis‐related marker. (B) Western blot analysis of ferroptosis‐associated proteins GPX4, ACSL4, FSP1, GCLM, SLC7A11, KEAP1 and NRF2 in tumour tissues; β‐actin served as a loading control (*n* = 3). (C–I) Densitometric quantification of protein expression levels using ImageJ for GPX4, FSP1, ACSL4, GCLM, SLC7A11, NRF2 and KEAP1 (*n* = 3). (J–N) Measurement of oxidative stress markers in tumour tissues, including MDA (J), SOD (K), GSH‐Px (L), level of GSH (M), GSH/GSSG ratio (N). Data are presented as mean ± SD. *p < 0.05, **p < 0.01, ***p < 0.001.

Histopathological analysis was conducted to further assess RSL3's impact on tumour progression. H&E staining revealed that RSL3 increased necrosis and cell death in tumour tissues compared to the MAE group (Figure 6M). TUNEL staining showed a significant increase in apoptotic (TUNEL‐positive) cells following RSL3 treatment, indicating enhanced apoptosis. IHC analysis demonstrated that RSL3 significantly downregulated the expression of Ki67 and GPX4 (Figure 7A), suggesting reduced proliferation and ferroptosis suppression. Western blot analysis supported these findings, showing that RSL3 decreased GPX4, FSP1, GCLM, SLC7A11 and NRF2 expression, while significantly upregulating ACSL4 and KEAP1 levels (*p <* 0.01; Figure 7B–I), a key enzyme involved in ferroptosis induction. To further investigate oxidative stress alterations in tumour tissues, we measured multiple key indicators, including MDA, SOD, GSH‐Px, total GPx, reduced GSH, the GSH/GSSG ratio and intracellular ROS levels. Compared with the MAE group, RSL3 treatment markedly elevated MDA levels, while significantly decreasing the activities of antioxidant enzymes SOD and GSH‐Px, as well as the levels of reduced GSH and the GSH/GSSG ratio (*p <* 0.01; Figure 7J–N). These findings indicate that RSL3 disrupts the antioxidant defence system and enhances oxidative stress in tumour tissues. Collectively, these results suggest that RSL3 counteracts the tumour‐promoting effects of CEVs by reactivating ferroptosis through the modulation of oxidative stress.

## 4 Discussion

Extracellular vesicles, as biologically active constituents of bacterial secretomes characterized by lipid bilayer‐enclosed nanostructures, have been extensively implicated in disease pathogenesis (Sheta et al. 2023). Our prior studies have revealed a positive correlation between the colonization abundance of *C. acnes* and the progression of EOC, with confirmation in a mouse model of EOC that *C. acnes* promotes tumour progression (Huang et al. 2022). However, the specific effector molecules and underlying mechanisms by which *C. acnes* contributes to EOC development have remained unclear.

Firstly, to identify the functional determinants by which *C. acnes* promotes the progression of EOC, we isolated the culture supernatant and heat‐inactivated bacterial cells of *C. acnes* and co‐cultured them separately with ID8 cells. Notably, the culture supernatant of *C. acnes* significantly enhanced ID8 cell proliferation, colony formation, migration, and invasion while suppressing apoptosis, suggesting that soluble secreted factors may serve as key mediators of its tumour‐promoting effect. Given the critical role of EVs in bacterial‐mediated diseases, we hypothesized that CEVs serve as critical mediators driving EOC progression (Chen, Gao, et al. 2024; Xu et al. 2024). Following isolation and purification using standardized protocols, transmission electron microscopy and particle size analysis confirmed the characteristic vesicular morphology of CEVs. In vitro functional assays demonstrated that CEVs largely recapitulated the pro‐proliferative, pro‐migratory and anti‐apoptotic effects of the bacterial culture supernatant. Consistently, in vivo CEVs treatment induced tumour‐promoting phenotypes comparable to those of *C. acnes*. Although a trend toward stronger effects was observed in the CEVs‐treated group, these differences did not reach statistical significance. Overall, this consistent pattern supports a contributory role of CEVs in *C. acnes*‐driven EOC progression and suggests that they may act as key effectors in both in vitro and in vivo settings. This phenomenon may, at least in part, be explained by differences in EVs exposure between EVs‐treated and bacteria‐associated conditions. The CEVs‐treated group received a defined and concentrated dose, whereas EV exposure in bacteria‐associated settings depends on bacterial colonization, survival and metabolic activity, which are subject to host regulation and may limit EVs production and delivery, resulting in lower or more variable exposure. In addition, purified CEVs are immediately bioavailable, whereas EVs production in the presence of live bacteria occurs progressively, potentially delaying their effects. Meanwhile, intact bacteria can trigger host immune responses and release non‐EVs components (e.g., metabolites and structural molecules), which may partially counteract or confound EVs‐mediated effects. In contrast, isolated CEVs represent a relatively enriched and uniform subset of bioactive cargo, leading to more consistent phenotypic outcomes. Collectively, these factors may account for the comparable or, in some cases, stronger effects observed in the CEVs‐treated group. Importantly, this finding is not an isolated case but is consistent with reports from other tumour‐associated bacteria (Xiang et al. 2024). Previous studies have demonstrated that extracellular vesicles from *Fusobacterium nucleatum* promote colorectal cancer progression through activation of host inflammatory pathways (Liu, Akbar, et al. 2024), while outer membrane vesicles from *Helicobacter pylori* carrying CagA facilitate gastric epithelial tumorigenesis (Díaz‐Garrido et al. 2021). These observations suggest that bacterial vesicles from diverse species may modulate host cell signalling and contribute to tumour development across different cancer types (Jin et al. 2025; Li et al. 2025). However, recent studies have also suggested that extracellular vesicles may exert beneficial or therapeutic effects under specific conditions. For example, Arce et al. reported that vesicles can enhance treatment responses (Arce‐Rodríguez et al. 2026). These findings are not necessarily contradictory to our results, but rather reflect the context‐dependent nature of vesicle‐mediated effects. The functional outcomes of extracellular vesicles likely depend on their origin and molecular cargo. In contrast, vesicles derived from tumour‐associated microbiota, such as *C. acnes*, may promote tumour progression by modulating redox homeostasis and suppressing ferroptosis.

Mechanistic analyses, including transcriptomics, qRT‐PCR and immunoblotting, demonstrated that CEVs induce extensive transcriptional reprogramming in ID8 cells, particularly affecting redox homeostasis and ferroptosis regulation. Specifically, CEVs significantly upregulated GPX4, FSP1, GCLM, SLC7A11 and NRF2, while downregulating ACSL4 and KEAP1, thereby enhancing GSH synthesis, increasing GPX4 activity and reducing lipid peroxidation and intracellular ROS levels. These results indicate that CEVs suppress ferroptosis through two complementary axes: on one hand, CEVs activate the KEAP1‐NRF2‐SLC7A11‐GCLM‐GPX4‐ROS‐ferroptosis axis to enhance cellular antioxidant capacity. As a negative regulator of NRF2, downregulation of KEAP1 promotes NRF2 nuclear translocation and transcriptional activation of downstream antioxidant genes (Kopacz et al. 2020; Chen, Xiao, et al. 2024). SLC7A11, as the core subunit of system Xc^−^, mediates cystine import to support GSH synthesis, while GCLM contributes to the rate‐limiting step of GSH production (Zheng and Conrad 2020; Lee and Roh 2022). The increased GSH pool further enhances GPX4 activity, detoxifying lipid peroxides and thereby blocking ferroptosis (Maiorino et al. 2018; Xia et al. 2024). On the other hand, CEVs modulate the KEAP1‐NRF2‐SLC7A11‐ACSL4‐ROS‐ferroptosis axis to reduce cellular sensitivity to lipid peroxide substrates. ACSL4 catalyses the esterification of polyunsaturated fatty acids into membrane phospholipids, sensitizing cells to lipid peroxidation and ferroptosis (Ding et al. 2023). Downregulation of ACSL4 by CEVs likely reduces the pool of oxidizable PUFA‐PE substrates, further suppressing lipid peroxidation and decreasing ferroptotic susceptibility. By simultaneously enhancing GSH/GPX4‐dependent detoxification of lipid peroxides and reducing ferroptosis‐priming lipid substrates, this dual regulatory mechanism provides a significant survival advantage to EOC cells. Notably, although direct evidence for CEVs regulating ferroptosis is limited, studies on extracellular vesicles from other bacteria support this concept (Liao et al. 2025; Yang et al. 2025). For example, extracellular vesicles from *Lactiplantibacillus plantarum* (LCEVs) inhibit macrophage ferroptosis by delivering miRNA cbn‐let‐7 to target ACSL4, alleviating tissue damage, while *Lactobacillus amylovorus* –derived vesicles mitigate oxidative stress‐induced ferroptosis in mammary epithelial cells by transferring oleic acid, enhancing cellular resilience (Zhang et al. 2025; Zhao et al. 2025). These studies, together with our findings, highlight the potential generality of bacterial extracellular vesicles in modulating ferroptosis and redox homeostasis in host cells, underscoring the novelty of our observations in ovarian cancer.

Importantly, the functional relevance of ferroptosis suppression was validated by pharmacological intervention with RSL3, a GPX4 inhibitor (Herrick et al. 2025). RSL3 treatment largely abrogated the tumour‐promoting effects of CEVs, reversed the upregulation of NRF2‐SLC7A11‐GCLM‐GPX4, and restored ACSL4 expression, concomitant with increased ROS and MDA levels, impaired redox balance, and enhanced ferroptotic cell death. These findings underscore ferroptosis as a critical downstream effector in CEVs‐mediated EOC progression.

In summary, our study provides the direct evidence that tumour‐intrinsic bacterial extracellular vesicles can regulate ferroptosis in ovarian cancer cells in vitro and in vivo, revealing a novel mechanism by which microbiota drive ovarian cancer progression. We found that CEVs coordinate the activation of the KEAP1‐NRF2‐SLC7A11‐GCLM‐GPX4‐ROS signalling axis to enhance cellular antioxidant capacity, while concurrently modulating the KEAP1‐NRF2‐SLC7A11‐ACSL4‐ROS axis to reduce the sensitivity of lipid peroxide substrates, thereby synergistically suppressing ferroptosis and promoting EOC cell survival and resistance to apoptosis. Despite these important findings, several limitations remain. First, the precise upstream molecular mechanism by which CEVs regulate the KEAP1/NRF2 pathway has not yet been fully elucidated. Because CEVs simultaneously carry proteins, non‐coding RNAs, lipids and metabolites (Kalluri and LeBleu 2020), the current experimental approaches are insufficient to determine whether their modulation of the KEAP1/NRF2 axis is mediated directly or occurs indirectly through upstream signalling pathways. Second, the in vivo attribution of CEVs‐specific effects still requires further validation. Although comparisons among PBS, bacterial supernatant, and purified CEVs support an important functional contribution of CEVs, and the additional qPCR results indicate that *C. acnes* signals are detectable in tumour tissues following administration, the current evidence is still insufficient to fully distinguish the relative contributions of exogenously administered bacteria, bacteria‐derived EVs, and other non‐EV bacterial components to the observed in vivo phenotypes. Therefore, future studies incorporating more stringent EVs purification strategies, EVs‐depleted controls, bacterial tracking, and quantitative assessment of EV exposure will be necessary to improve the precision of mechanistic attribution. Finally, the biological effects mediated by CEVs remain to be further explored within the broader context of the tumour microenvironment. Our current findings suggest that ferroptosis is more likely to be the predominant regulated cell death pathway affected by CEVs; however, the relative contributions of different RCD pathways still require more systematic evaluation. In addition, ovarian cancer progression involves complex interactions among cancer cells, immune cells, and stromal components (MacFawn et al. 2024). Future studies should further investigate whether CEVs‐mediated suppression of ferroptosis also influences the tumour immune microenvironment and the response to immunotherapy.

## 5 Conclusions

In summary, this study identifies CEVs as key mediators of tumour–microbe interactions in epithelial ovarian cancer. Mechanistically, CEVs promote tumour progression by activating the KEAP1–NRF2 axis, leading to suppression of ferroptosis through enhanced glutathione metabolism, increased GPX4 activity, and reduced lipid peroxidation and ROS levels. Notably, pharmacological induction of ferroptosis with RSL3 effectively abrogates the tumour‐promoting effects of CEVs both in vitro and in vivo, underscoring ferroptosis inhibition as a central mechanism. Collectively, these findings highlight bacterial extracellular vesicles as important drivers of tumour progression and suggest that targeting vesicle‐mediated signalling or restoring ferroptotic sensitivity may offer potential therapeutic avenues for epithelial ovarian cancer.

## Author Contributions

**Qifa Huang:** writing – original draft, investigation, data curation, formal analysis. **Qi Chen:** formal analysis, writing – review and editing. **Wenjie Xiong:** formal analysis, writing – review and editing. **Yuexi Sun:** writing – review and editing. **Yuxiong Huang:** writing – review and editing. **Ang Dai:** writing – review and editing. **Jianying Chen:** writing – review and editing. **Xue Wu:** writing – review and editing. **Ying Jiang:** formal analysis. **Fen Wei:** formal analysis. **Qi Chen:** conceptualization, supervision, writing – review and editing. **Tingtao Chen:** conceptualization, supervision, funding acquisition, writing – review and editing.

## Funding

This work was supported by the National Natural Science Foundation of China (Grant Nos. 82460528 and 82260507 to Q.C.) and the Jiangxi Provincial Postgraduate Innovation Fund (Grant No. YC2024‐B048 to Q.H.).

## Ethics Statement

All animal experiments were performed in compliance with the ethical guidelines and regulatory standards approved by the Institutional Animal Care and Use Committee of Nanchang University, China (Approval No. NCULAE‐20221031068).

## Conflicts of Interest

The authors declare no conflicts of interest.

## Supporting information


**Figure S1:** Identification and characterization of pro‐tumorigenic components in *C. acnes*. (A) ID8 cells were treated with PBS (P), *C. acnes* bacterium (B), or *C. acnes* culture supernatant (S), and cell proliferation was monitored at 6, 24, and 48 h using manual cell counting to assess the effects on cell growth. (B) ID8 cells were exposed to increasing concentrations of CEVs, and proliferation was quantified at 6, 24 and 48 h using the CCK‐8 assay. (C) Representative flow cytometry plots showing (top row) FSC‐A vs. SSC‐A gating for viable cells, (middle row) FSC‐H vs. FSC‐A gating for singlet discrimination, and (bottom row) Annexin V/PI double staining for apoptosis assessment. Data are presented as mean ± SD. *p < 0.05, **p < 0.01, ***p < 0.001, ****p < 0.0001.
**Figure S2:** Transcriptomic alterations and pathway enrichment in ID8 cells following CEVs treatment. (A) Representative flow cytometry plots showing (top row) FSC‐A vs. SSC‐A gating for viable cell populations, (middle row) FSC‐H vs. FSC‐A gating for singlet discrimination, and (bottom row) Annexin V/PI double staining for apoptosis analysis. (B) Principal component analysis (PCA) showing clear separation between PBS‐treated (P) and CEVs‐treated (E) groups, indicating substantial differences in global gene expression profiles. (C) Summary of differentially expressed genes, with 1310 upregulated and 369 downregulated genes in the CEVs‐treated group compared with PBS controls (*p* < 0.05, fold change ≥ 2). (D) Heatmap illustrating expression patterns of ferroptosis‐related genes significantly altered by CEVs treatment. (E) Heatmap illustrating expression patterns of apoptosis‐related genes significantly altered by CEVs treatment. (F) KEGG pathway analysis showing significant upregulation of glutathione metabolism and fluid shear stress/atherosclerosis pathways in the CEVs‐treated group. (G) DHE staining was performed to detect intracellular ROS levels, and fluorescence intensity reflects superoxide (O2−) accumulation (scale bar = 50 μm).
**Figure S3:** Pharmacological inhibition of NRF2 attenuates the CEV‐induced anti‐ferroptotic gene programme in ID8 cells. (A) Schematic illustration of the experimental design. ID8 cells were treated with PBS (P), *C. acnes*–derived extracellular vesicles (CEVs; E), ML385 (M), or CEVs plus ML385 (EM). (B–G) Relative mRNA expression levels of GPX4 (B), FSP1 (C), ACSL4 (D), GCLM (E), SLC7A11 (F) and NRF2 (G), determined by RT‐qPCR. (H) Representative Western Blot images showing protein expression of GPX4, FSP1, ACSL4, GCLM, SLC7A11 and NRF2, with β‐actin as the loading control. (I–N) Quantification of protein levels of GPX4 (I), FSP1 (J), ACSL4 (K), GCLM (L), SLC7A11 (M) and NRF2 (N), normalized to β‐actin. Data are presented as mean ± SD from three independent experiments. Statistical significance was analysed by one‐way ANOVA with appropriate post hoc multiple‐comparison testing. *p < 0.05, **p < 0.01, ***p < 0.001.
**Figure S4:** Induction of ferroptosis attenuates CEVs‐induced EOC progression in vitro. (A) Schematic overview of the in vitro experimental workflow. (B) CCK‐8 assay showing that CEVs treatment significantly promoted ID8 cell proliferation, whereas co‐treatment with RSL3 largely reversed this effect. (C, D) Representative images and quantitative analysis of wound‐healing assays demonstrating that CEVs enhanced cell migration, which was markedly attenuated by RSL3 co‐treatment. (E) Representative flow cytometry plots showing (top row) FSC‐A vs. SSC‐A gating for viable cells, (middle row) FSC‐H vs. FSC‐A gating for singlet discrimination, and (bottom row) Annexin V/PI double staining for apoptosis assessment. (F) Representative images of DHE staining showing decreased intracellular ROS levels (scale bar = 50 μm). Data are presented as mean ± SD; *p < 0.05, **p < 0.01, ***p < 0.001.
**Figure S5:** Extracellular vesicles derived from *Cutibacterium acnes* enhance EOC advancement by modulating ferroptosis. (A) Relative abundance of C. acnes in tumour tissues determined by qPCR (*n* = 5). (B) Representative immunohistochemical staining of 4‐HNE in tumour sections. Positive staining is indicated by brown coloration, with haematoxylin counterstaining (blue). Scale bars, 25 μm. (C) Quantification of 4‐HNE–positive cells in each group (*n* = 3). Data are presented as mean ± SD; *p < 0.05, **p < 0.01, ***p < 0.001.
**Table S1:** Cell line metadata and authentication status.
**Table S2:** Chemicals information.
**Table S3:** The grouping treatment of animal.
**Table S4:** The information of primers.
**Table S5:** The information of each specific antibody.

## Data Availability

The RNA‐seq data generated in this study have been deposited in the NCBI Sequence Read Archive (SRA) under BioProject accession number PRJNA1338457 and are publicly available at https://www.ncbi.nlm.nih.gov/bioproject/PRJNA1338457. All other data supporting the findings of this study are included in the Supporting Information or are available from the corresponding author upon reasonable request.
