# Supplementary material for: Cutibacterium acnes–Derived Extracellular Vesicles Promote Epithelial Ovarian Cancer Progression by Activating the KEAP1–NRF2 Antioxidant Pathway to Suppress Ferroptosis
Source: Microb Biotechnol. 2026 May 11;19(5):e70373. doi: 10.1111/1751-7915.70373 (PMC13160924; doi:10.1111/1751-7915.70373)
Supplement: Supplementary file 1 — Figure S1: Identification and characterization of pro‐tumorigenic components in C. acnes. (A) ID8 cells were treated with PBS (P), C. acnes bacterium (B), or C. acnes culture supernatant (S), and cell proliferation was monitored at 6, 24, and 48 h using manual cell counting to assess the effects on cell growth. (B) ID8 cells were exposed to increasing concentrations of CEVs, and proliferation was quantified at 6, 24 and 48 h using the CCK‐8 assay. (C) Representative flow cytometry plots showing (top row) FSC‐A vs. SSC‐A gating for viable cells, (middle row) FSC‐H vs. FSC‐A gating for singlet discrimination, and (bottom row) Annexin V/PI double staining for apoptosis assessment. Data are presented as mean ± SD. *p < 0.05, **p < 0.01, ***p < 0.001, ****p < 0.0001. Figure S2: Transcriptomic alterations and pathway enrichment in ID8 cells following CEVs treatment. (A) Representative flow cytometry plots showing (top row) FSC‐A vs. SSC‐A gating for viable cell populations, (middle row) FSC‐H vs. FSC‐A gating for singlet discrimination, and (bottom row) Annexin V/PI double staining for apoptosis analysis. (B) Principal component analysis (PCA) showing clear separation between PBS‐treated (P) and CEVs‐treated (E) groups, indicating substantial differences in global gene expression profiles. (C) Summary of differentially expressed genes, with 1310 upregulated and 369 downregulated genes in the CEVs‐treated group compared with PBS controls (p < 0.05, fold change ≥ 2). (D) Heatmap illustrating expression patterns of ferroptosis‐related genes significantly altered by CEVs treatment. (E) Heatmap illustrating expression patterns of apoptosis‐related genes significantly altered by CEVs treatment. (F) KEGG pathway analysis showing significant upregulation of glutathione metabolism and fluid shear stress/atherosclerosis pathways in the CEVs‐treated group. (G) DHE staining was performed to detect intracellular ROS levels, and fluorescence intensity reflects superoxide (O2−) accumula [file MBT2-19-e70373-s001.docx]

***Cutibacterium acnes*–derived extracellular vesicles promote epithelial ovarian cancer progression by activating the KEAP1–NRF2 antioxidant pathway to suppress ferroptosis**

Qifa Huang^1,2,3^ | Qi Chen^4^ | Wenjie Xiong^5^ | Yuexi Sun^5,6,7^ | Yuxiong Huang^5,6,7^ | Ang Dai^1,2^ | Jianying Chen^5,6^ | Xue Wu^1^ | Ying Jiang^1^ | Fen Wei^1^ | Qi Chen^1,2^ | Tingtao Chen^1,5,6^

^1^Department of Obstetrics and Gynecology, The Second Affiliated Hospital, Jiangxi Medical College, Nanchang University, Nanchang, Jiangxi, China | ^2^Jiangxi Key Laboratory of Molecular Medicine, The Second Affiliated Hospital of Nanchang University, Nanchang University, Nanchang, Jiangxi, China | ^3^School of Clinical Medicine, Jiangxi University of Chinese Medicine, Nanchang, Jiangxi, China | ^4^Department of Radiology, Nanchang People's Hospital, Affiliated Hospital of Nanchang Medical College, Nanchang, Jiangxi, China | ^5^Jiangxi Province Key Laboratory of Bioengineering Drugs, School of Pharmacy, Nanchang University, Nanchang, Jiangxi, China | ^6^National Engineering Research Center for Bioengineering Drugs and the Technologies, Institute of Translational Medicine, Jiangxi Medical College, Nanchang University, Nanchang, Jiangxi, China | ^7^Queen Mary School, Jiangxi Medical College, Nanchang University, Nanchang, Jiangxi, China

**Correspondence:** Qi Chen ([chenqiyangbai@126.com)](mailto:chenqiyangbai@126.com;) | Tingtao Chen ([chentingtao1984@163.com](mailto:chentingtao1984@163.com); [chengtingtao@ncu.edu.cn)](mailto:chengtingtao@ncu.edu.cn))

**Supplementary figures and tables**
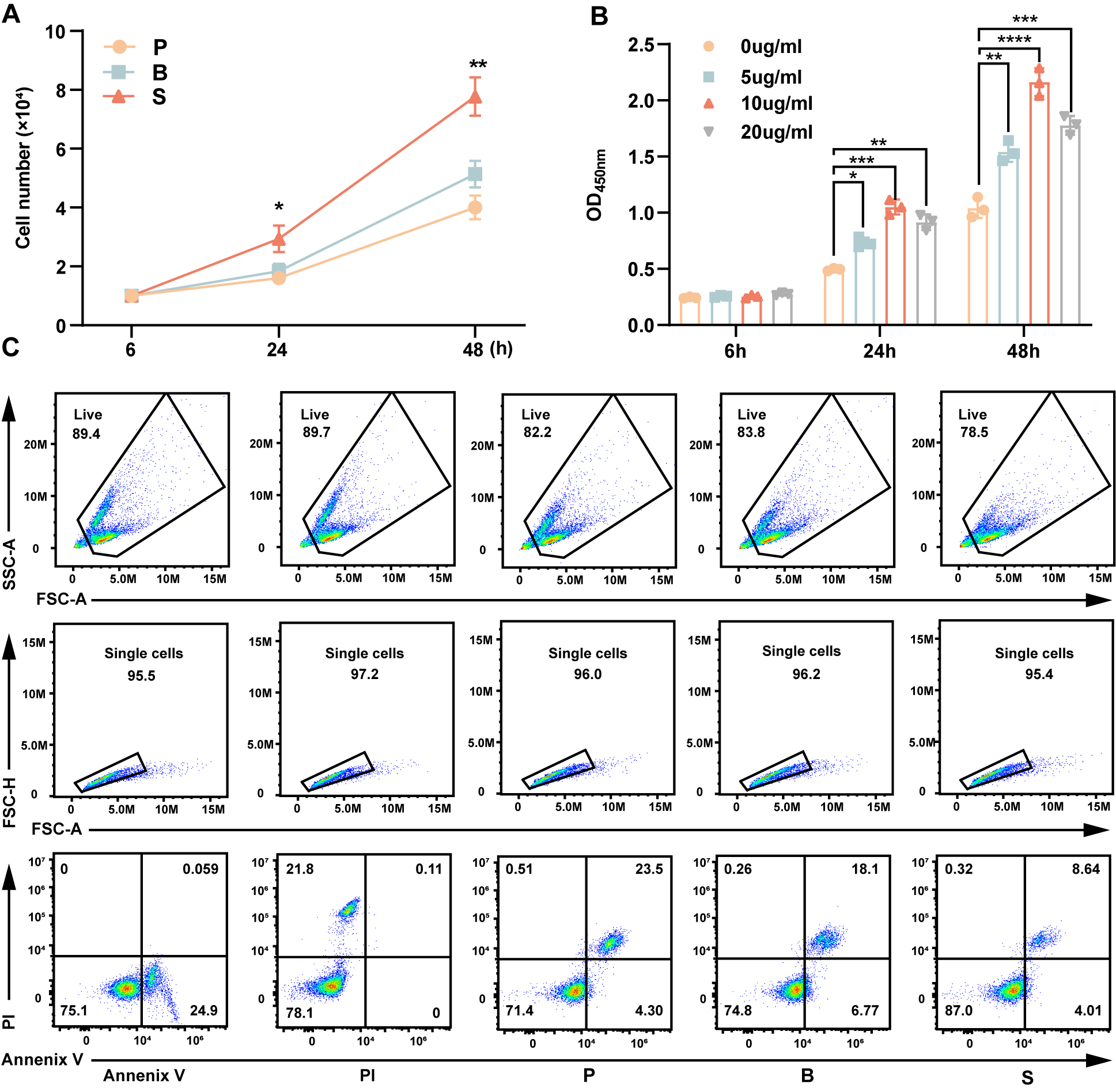


**Supplementary Figure S1.** Identification and characterization of pro-tumorigenic components in *C. acnes.* (A) ID8 cells were treated with PBS (P), *C. acnes bacterium* (B), or *C. acnes* culture supernatant (S), and cell proliferation was monitored at 6, 24, and 48 hours using manual cell counting to assess the effects on cell growth. (B) ID8 cells were exposed to increasing concentrations of CEVs, and proliferation was quantified at 6, 24, and 48 hours using the CCK-8 assay. (C) Representative flow cytometry plots showing (top row) FSC-A vs. SSC-A gating for viable cells, (middle row) FSC-H vs. FSC-A gating for singlet discrimination, and (bottom row) Annexin V/PI double staining for apoptosis assessment. Data are presented as mean ± SD. **p* < 0.05, ***p* < 0.01, ****p* < 0.001, *****p* < 0.0001.


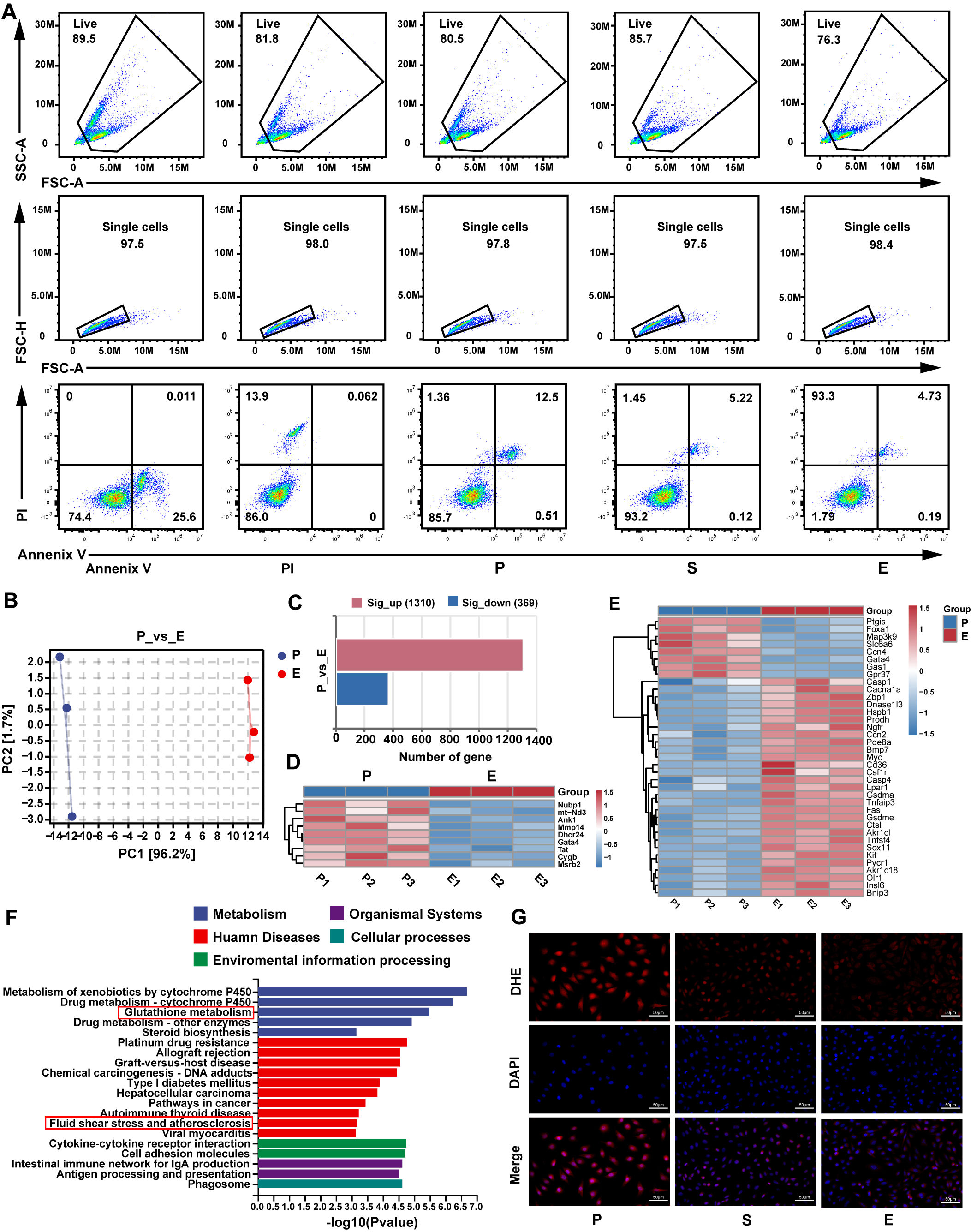


**Supplementary Figure S2.** Transcriptomic alterations and pathway enrichment in ID8 cells following CEVs treatment. (A) Representative flow cytometry plots showing (top row) FSC-A vs. SSC-A gating for viable cell populations, (middle row) FSC-H vs. FSC-A gating for singlet discrimination, and (bottom row) Annexin V/PI double staining for apoptosis analysis. (B) Principal component analysis (PCA) showing clear separation between PBS-treated (P) and CEVs-treated (E) groups, indicating substantial differences in global gene expression profiles. (C) Summary of differentially expressed genes, with 1,310 upregulated and 369 downregulated genes in the CEVs-treated group compared with PBS controls (*p* < 0.05, fold change ≥ 2). (D) Heatmap illustrating expression patterns of ferroptosis-related genes significantly altered by CEVs treatment. (E) Heatmap illustrating expression patterns of apoptosis-related genes significantly altered by CEVs treatment. (F) KEGG pathway analysis showing significant upregulation of glutathione metabolism and fluid shear stress/atherosclerosis pathways in the CEVs-treated group. (G) DHE staining was performed to detect intracellular ROS levels, and fluorescence intensity reflects superoxide (O_₂_⁻) accumulation (scale bar = 50 μm).


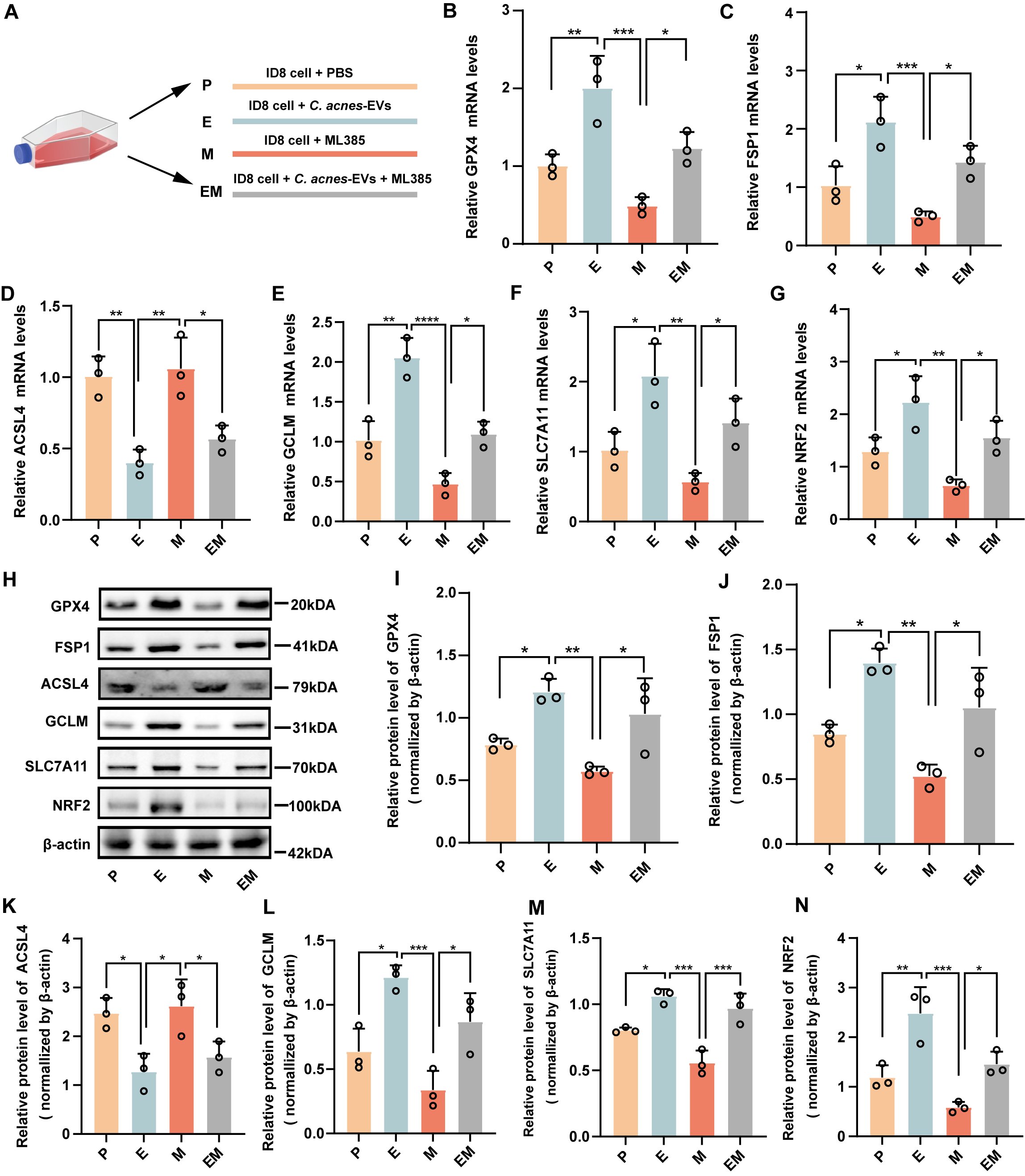


**Supplementary Figure S3.** Pharmacological inhibition of NRF2 attenuates the CEV-induced anti-ferroptotic gene program in ID8 cells. (A) Schematic illustration of the experimental design. ID8 cells were treated with PBS (P), *C. acnes*-derived extracellular vesicles (CEVs; E), ML385 (M), or CEVs plus ML385 (EM). (B–G) Relative mRNA expression levels of GPX4 (B), FSP1 (C), ACSL4 (D), GCLM (E), SLC7A11 (F), and NRF2 (G), determined by RT-qPCR. (H) Representative Western Blot images showing protein expression of GPX4, FSP1, ACSL4, GCLM, SLC7A11, and NRF2, with β-actin as the loading control. (I–N) Quantification of protein levels of GPX4 (I), FSP1 (J), ACSL4 (K), GCLM (L), SLC7A11 (M), and NRF2 (N), normalized to β-actin. Data are presented as mean ± SD from three independent experiments. Statistical significance was analyzed by one-way ANOVA with appropriate post hoc multiple-comparison testing. **p* < 0.05, ***p* < 0.01, ****p* < 0.001.


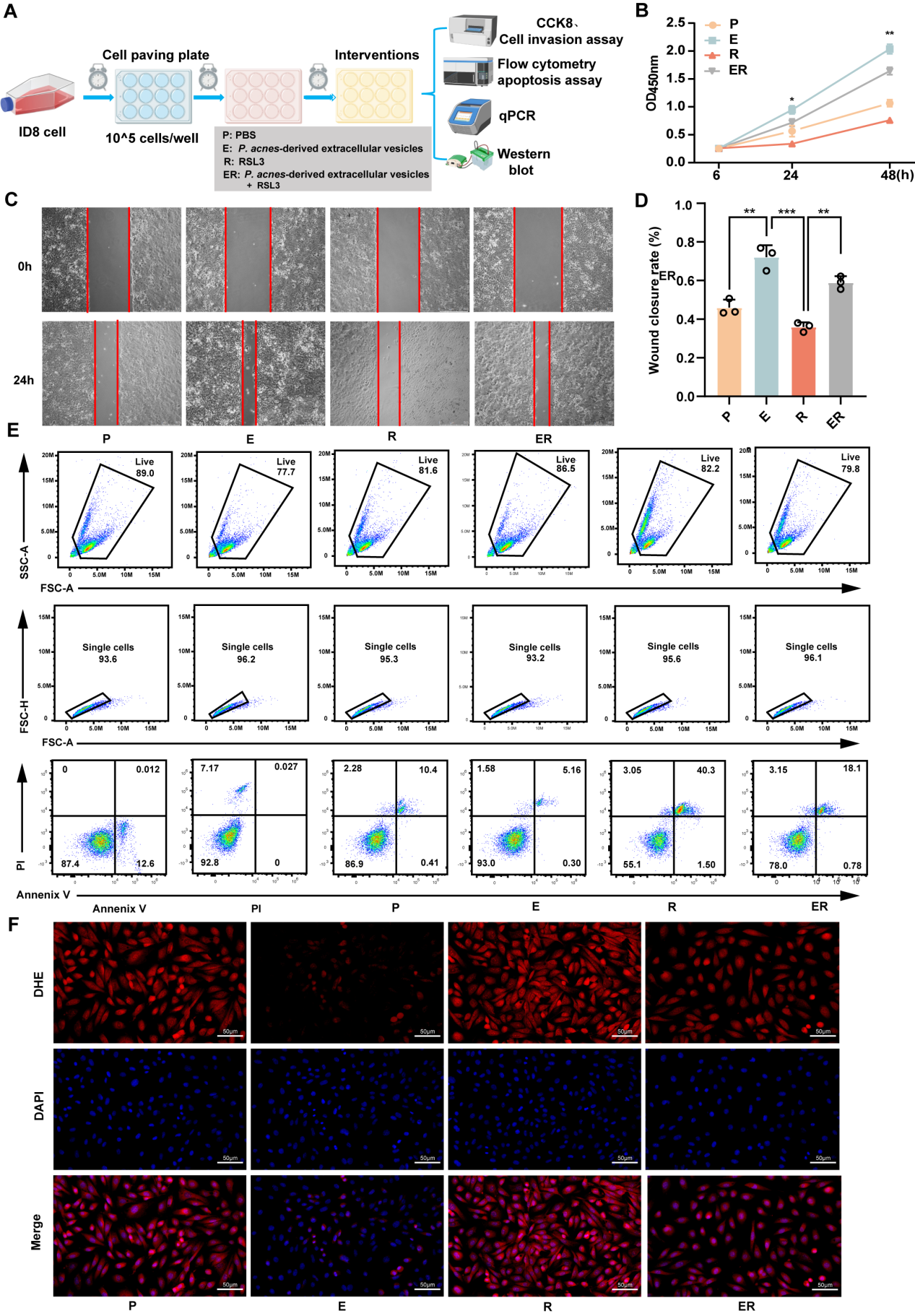


**Supplementary Figure S4.** Induction of ferroptosis attenuates CEVs-induced EOC progression *in vitro.* (A) Schematic overview of the *in vitro* experimental workflow. (B) CCK-8 assay showing that CEVs treatment significantly promoted ID8 cell proliferation, whereas co-treatment with RSL3 largely reversed this effect. (C–D) Representative images and quantitative analysis of wound-healing assays demonstrating that CEVs enhanced cell migration, which was markedly attenuated by RSL3 co-treatment. (E) Representative flow cytometry plots showing (top row) FSC-A vs. SSC-A gating for viable cells, (middle row) FSC-H vs. FSC-A gating for singlet discrimination, and (bottom row) Annexin V/PI double staining for apoptosis assessment. (F) Representative images of DHE staining showing decreased intracellular ROS levels (scale bar = 50 μm). Data are presented as mean ± SD; **p* < 0.05, ***p* < 0.01, ****p* < 0.001.


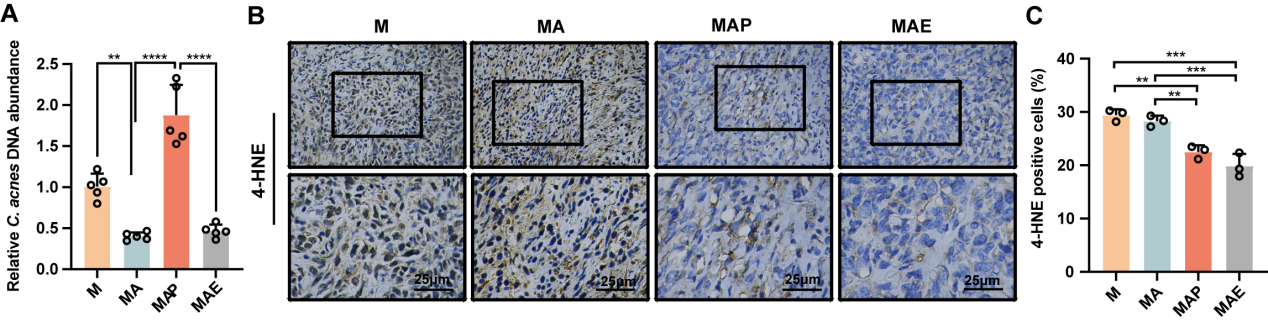


**Supplementary Figure S5.** Extracellular vesicles derived from *Cutibacterium acnes* enhance EOC advancement by modulating ferroptosis. (A) Relative abundance of *C. acnes* in tumor tissues determined by qPCR (n = 5). (B) Representative immunohistochemical staining of 4-HNE in tumor sections. Positive staining is indicated by brown coloration, with hematoxylin counterstaining (blue). Scale bars, 25 μm. (C) Quantification of 4-HNE–positive cells in each group (n = 3). Data are presented as mean ± SD; **p* < 0.05, ***p* < 0.01, ****p* < 0.001.

Table S1. Cell Line Metadata and Authentication Status

| **Feature** | **ID8** |
| --- | --- |
| Official Name | ID8 |
| RRID | CVCL_IU14 |
| Species | *Mus musculus* (mouse) |
| Sex | Female |
| Tissue of Origin | Ovarian surface epithelium |
| Supplier | Shanghai Fuxiang Biotechnology |
| Catalog Number | XF1030 |
| Obtained Date | May 2019 |
| Authentication Method | STR profiling(20 loci) |
| % Match Result | 100% |
| Mycoplasma Status | Negative |

Table S2. Chemicals information

| **Chemicals** |  |  |
| --- | --- | --- |
| Brain Heart Infusion (BHI) | CHINOOK | Cat# CN230975 |
| Agar | Fisher BD | Cat# DF0140-01-0 |
| Ampicillin | Sangon Biotech | - Cat# A610028-0025 |
| Vancomycin hydrochloride | Sangon Biotech | - Cat# A600983-0001 |
| Neomycin trisulfate salt hydrate | Sangon Biotech | - Cat# A610366-0025 |
| Metronidazole | Sangon Biotech | - Cat# A600633-0025 |
| ML385 | MedChem Express | Cat# HY-100523 |
| RSL3 | MedChem Express | Cat# [1219810-16-8](https://www.medchemexpress.cn/search.html?q=1219810-16-8&ft=&fa=&fp=) |
| DMEM | Gibco^TM^ | Cat# 11-995-065 |
| FBS | Gibco^TM^ | Cat# A5669701 |
| PBS | Gibco^TM^ | Cat# 00-3002 |
| DAPI | Sigma Aldrich | Cat# D9542 |

Table S3.The grouping treatment of animal

|  | **Antibiotic**  **mixture** | **Intratumoral**  ***C. acnes* injection** | **Intratumoral CEVs injection** | **RSL3 injection** |
| --- | --- | --- | --- | --- |
| Group M | − | − | − | − |
| Group MA | + | − | − | − |
| Group MAP | + | + | − | − |
| Group MAE | + | − | + | − |
| Group MAPR | + | + | − | + |
| Group MAER | + | − | + | + |

Table S4. The information of primers

| **Name** | **Forward primer sequences** | **Reverse primer sequences** |
| --- | --- | --- |
| β-actin | 5’-CTACCTCATGAAGATCCTGACC-3’ | 5’-CACAGCTTCTCTTTGATGTCAC-3’ |
| NQO1 | 5’-GCCGAACACAAGAAGCTGGAAG-3’ | 5’-GGCAAATCCTGCTACGAGCACT-3’ |
| SLC7A11 | 5’-CCTCTGACGATGGTGATGCTCTTC-3’ | 5’-GGTGCTGAATGGGTCCGAGTAAAG-3’ |
| GCLM | 5’-AGGAGCTTCGGGACTGTATCC-3’ | 5’-GGGACATGGTGCATTCCAAAA-3’ |
| GPX4 | 5’-CAGGAGCCAGGAAGTAATCAAG -3’ | 5’-GCATCGTCCCCATTTACACAG -3’ |
| CAT | 5’-CGGCACATGAATGGCTATGGATC-3’ | 5’-AAGCCTTCCTGCCTCTCCAACA-3’ |
| KEAP1 | 5’-ATCCAGAGAGGAATGAGTGGCG-3’ | 5’-TCAACTGGTCCTGCCCATCGTA-3’ |
| NRF2 | 5’-GCATAGAGCAGGACATGGAGCAAG-3’ | 5’-ACTGATGGCAGCGGAGGAAGG-3’ |
| ACSL4 | 5’-ATTGGTCAGGGATATGGGCT-3’ | 5’-AGAGGAGCTCCAACTCTTCCA-3’ |
| FSP1 | 5’-CTAGGCCACAGAATTGAAAGATC-3’ | 5’-GTAGGTGGAAATTCTAGCATCAT-3’ |
| HO-1   1. *acnes* | 5’-GAGCCTGAATCGAGCAGAAC-3’  5’-GCGT GAGT GACG GTAAT GGGTA-3’ | 5’-CCTTCAAGGCCTCAGACAAA-3’  5’-TTCCGACGCGATCAACCA-3’ |

Table S5. The information of each specific antibody

| **Protein** | **Product Catalog** | **Brand** | **Dilution ratio** |
| --- | --- | --- | --- |
| Mouse anti-β-actin | 66009-1-Ig | Proteintech | 1:20000 |
| Rabbit anti-KI67 | 28074-1-AP | Proteintech | 1:2000 |
| Mouse anti-GPX4 | 67763-1-Ig | Proteintech | 1:1000 |
| Rabbit anti-NRF2 | 16396-1-AP | Proteintech | 1:2000 |
| Rabbit anti-KEAP1 | 10503-2-AP | Proteintech | 1:2000 |
| Rabbit anti-ACSL4 | 22401-1-AP | Proteintech | 1:4000 |
| Rabbit anti-FSP1 | 20886-1-AP | Proteintech | 1:1000 |
| Rabbit anti-SLC7A11 | 26864-1-AP | Proteintech | 1:1000 |
| Mouse anti-GCLM | 66808-1-Ig | Proteintech | 1:2000 |
| HRP-conjugated Anti-Mouse IgG | SA00001-1 | Proteintech | 1:10000 |
| HRP-conjugated Anti-Rabbit IgG | SA00001-2 | Proteintech | 1:10000 |
